# Language Structure Is Partly Determined by Social Structure

**DOI:** 10.1371/journal.pone.0008559

**Published:** 2010-01-20

**Authors:** Gary Lupyan, Rick Dale

**Affiliations:** 1 Institute for Research on Cognitive Science and Center for Cognitive Neuroscience, University of Pennsylvania, Philadelphia, Pennsylvania, United States of America; 2 Department of Psychology, The University of Memphis, Memphis, Tennessee, United States of America; University of Utah, United States of America

## Abstract

**Background:**

Languages differ greatly both in their syntactic and morphological systems and in the social environments in which they exist. We challenge the view that language grammars are unrelated to social environments in which they are learned and used.

**Methodology/Principal Findings:**

We conducted a statistical analysis of >2,000 languages using a combination of demographic sources and the World Atlas of Language Structures— a database of structural language properties. We found strong relationships between linguistic factors related to morphological complexity, and demographic/socio-historical factors such as the number of language users, geographic spread, and degree of language contact. The analyses suggest that languages spoken by large groups have simpler inflectional morphology than languages spoken by smaller groups as measured on a variety of factors such as case systems and complexity of conjugations. Additionally, languages spoken by large groups are much more likely to use lexical strategies in place of inflectional morphology to encode evidentiality, negation, aspect, and possession. Our findings indicate that just as biological organisms are shaped by ecological niches, language structures appear to adapt to the environment (niche) in which they are being learned and used. As adults learn a language, features that are difficult for them to acquire, are less likely to be passed on to subsequent learners. Languages used for communication in large groups that include adult learners appear to have been subjected to such selection. Conversely, the morphological complexity common to languages used in small groups increases redundancy which may facilitate language learning by infants.

**Conclusions/Significance:**

We hypothesize that language structures are subjected to different evolutionary pressures in different social environments. Just as biological organisms are shaped by ecological niches, language structures appear to adapt to the environment (niche) in which they are being learned and used. The proposed *Linguistic Niche Hypothesis* has implications for answering the broad question of why languages differ in the way they do and makes empirical predictions regarding language acquisition capacities of children versus adults.

## Introduction

**Figure 1:**
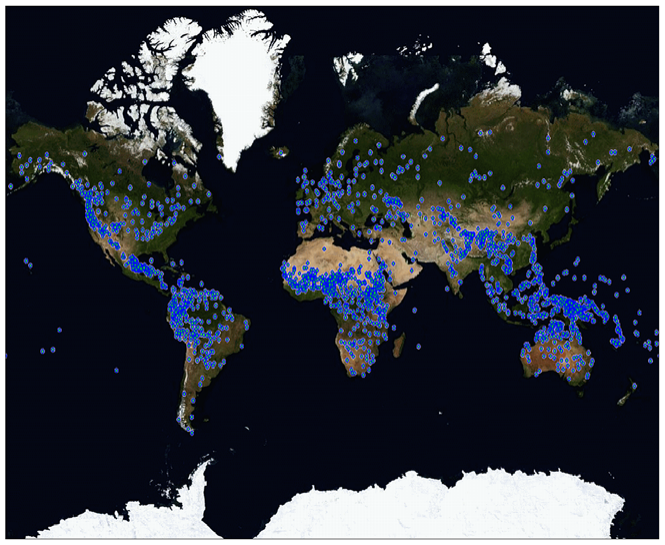
Geographic distribution of the 2,236 languages included in the present study.

Although the largest languages are spoken by millions of people spread over vast geographic areas, most languages are spoken by relatively few individuals over comparatively small areas. The median number of speakers for the 6,912 languages catalogued by the Ethnologue is only 7,000, compared to the mean of over 828,000 [1]. Similarly, for the 2,236 languages in our sample (Figure 1), the median area over which a language is spoken is about the size of Luxembourg or San Diego, California (948 km^2^). The mean area is about the size of Austria or the US state of Maryland (33,795 km^2^). Languages also differ dramatically in the proportion of individuals who speak the language natively (L1 speakers) to those who learned it later in life (L2 speakers) (Table S1). Although there are numerous counter-examples (Text S1), languages spoken by millions of people have a greater likelihood of coming into contact with other languages and of having numerous nonnative speakers compared to languages spoken by only a few thousand people. This is not surprising: a language spoken by more people is more likely to encompass a larger and more diverse area and include speakers from varying ethnic and linguistic backgrounds. Conversely, languages spoken by a thousand or even fewer individuals tend to be spoken in highly circumscribed locales (Text S2). Overall, languages with smaller speaker populations are more likely to be spoken by more socially cohesive groups [2] than languages that have millions of speakers.

Just as there are socio-historical and demographic differences among the world's languages, there are also vast differences among languages in morphology and syntax [3]. For example languages differ in the devices used to convey syntactic relations—who did what to whom. Some languages rely on a fixed word order (Subject-Verb-Object in the case of English), while other languages (e.g., German, Polish) allow much more flexibility in word order and rely on case markings to signal which noun fills the role of subject, object, etc. [4] More generally, languages differ in the amount of information conveyed through inflectional morphology compared to the amount of information conveyed through non-morphological devices such as word order and lexical constructions. For example, compare morphological marking of aspect in Russian “Ya *vy*pil chai” (I PERFECTIVE+drank tea), to the English lexical strategy, “I *finished drinking* the tea.” Some other domains exhibiting such differences between lexical and morphological strategies include tense, aspect, evidentiality, negation, plurality, and expressions of possibility.

Languages with richer morphological systems are said to be more overspecified [5]–[7]. For instance, of the languages that encode the past tense inflectionally, about 20% have past tenses that explicitly mark remoteness distinctions. For example Yagua, a language of Peru, has inflections that differentiate 5 levels of remoteness. A verb denoting an event that happened only a few hours ago takes the suffix –*jásiy*; an event that happened a day previous to the utterance requires a different suffix, *-jay*; an event that occurred a week to a month ago, a still different suffix, -*siy*, etc. [8]. Of course, languages without these grammatical distinctions can express them lexically, as in English: “I broke my foot a few years ago.” On the other hand, when semantic distinctions are encoded grammatically, speakers are generally obligated to make them [9], hence sentences concerning the past will have its remoteness specified even when it may not be relevant to the discourse. In the English example above, speakers have the option to omit remoteness information, but are obligated to express the grammatically encoded past tense (which leaves remoteness to context). In Mandarin or Thai, which express both tense and remoteness lexically, speakers have the option of omitting the past tense entirely. Of the 222 languages in our corpus for which tense information is available, 40% do not encode past tense inflectionally [10].

The degree and specificity of morphological encoding can reach astounding levels. For example, Karok—a language of N.W. California—has morphological suffixes for forms of containment *pa:θ-kirih* “throw into fire”, *pa:θ-kurih* “throw into water”, *pa:θ-ruprih* “throw in through a solid” (the affixes are unrelated to the lexemes for water, fire, etc.) [11]. Clearly, such elaboration does not arise from communicative necessity. Researchers have long been puzzled by the reasons why some languages abound in such overspecification, while others (sometimes closely related ones) eschew it. For example, in comparing English and German we find that where the surface structures of English and German contrast, English is less specified, leaving more to context [6], thus, “…German speakers are forced to make certain semantic distinctions which can regularly be left unspecified in English” [6], p. 28). For example, German obligatorily specifies the direction of motion in the place adverbs here/there/where. Compare: *hier/her*; *dort/hin*; *wo/wohin*. English can specify direction using to and from (“where *to*” versus “where *from*”), but such specification is optional and is generally omitted [12], [9]. Grammatical divergence between languages has been typically attributed to drift—as a population speaking an ancestral Germanic language splits into separate groups, their language gradually diverges with one branch becoming English and the other German [13]. Such accounts do not explain why English came to shed much of its morphology while German retained it.

Attempts to establish relationships between social and linguistic structure date back at least a century [14]–[16]; see [17] for a review. Recent work has provided some support for the idea that extralinguistic factors (e.g., degree of ecological risk) play a role in some aspects of language such as varying levels of linguistic diversity in different parts of the world [18], [19]. A number of researchers have investigated correlations between social environments and the phonological structure of languages [20]–[22] and, intriguingly, have also found correlations between physical aspects of the environment such as temperature, and phonological inventories [23], [24]. It has also been argued that the physical environment [25], and historical developments that impact language transmission can impact the syntactic and morphological structure of languages [2], [5], [26], [27].

Languages with histories of adult learning have been argued to be morphologically simpler, less redundant, and more regular/transparent [2], [7], [28]–[30]. This argument has been made most forcefully and convincingly for Creole languages [26], but it has been speculated that any situation in which a language is learned by a substantial number of adults it becomes simplified due to the “lousy language learning abilities of the human adult” [28]. The evidence for such linguistic simplification has been largely descriptive, consisting of selected examples and grammatical inventories of small numbers of languages [17], [14], [29], [7], [5] . Thus, at present, there is little convincing evidence of global relationships between linguistic structure and non-linguistic factors and limited theoretical frameworks within which to understand such relationships [e.g., 20 for the case of phonological inventories]. An additional limitation of previous work is that it fails to explain why morphological complexity and grammatical overspecification arise in the first place. That is, why aren't all languages as morphologically simple as those that have been argued to be heavily shaped by adult learning, e.g., English [12]?

The primary goal of the present work is to examine whether non-spurious relationships exist between social and linguistic structure by using large-scale demographic and linguistic databases. A secondary goal is to provide a tentative framework within which to understand the reported results—the *Linguistic Niche Hypothesis*—which provides a nomothetic account for understanding relationships between linguistic and social structure (Text S3).

In assessing the relationship between social and linguistic structure, it is useful to distinguish two main contexts (niches) in which languages are learned and used: the *exoteric* and the *esoteric* [2], [31]. The exoteric linguistic niche contains languages with large numbers of speakers, thus requiring these languages to serve as interfaces for communication between strangers. In reality the esoteric and exoteric niches form a continuum, and are represented as such in our analyses (see also Text S4). Speakers of languages in the exoteric niche compared to speakers of esoteric languages are more likely to (1) be nonnative speakers or have learned the language from nonnative speakers, and (2) use the language to speak to outsiders—individuals from different ethnic and/or linguistic backgrounds. The exoteric niche includes languages like English, Swahili, and Hindi, while the esoteric niche includes languages like Tatar, Elfdalian, and Algonquin.

## Results

To assess relationships between social and linguistic structure we constructed a dataset that combined social/demographic and typological information for 2,236 languages. Grammatical information was obtained from the World Atlas of Language Structures (WALS) [32]—a database of structural properties of language compiled from descriptive materials such as reference grammars. The full dataset was constructed by combining typological data from WALS with the following demographic variables: speaker population, geographic spread, and number of linguistic neighbors derived from Ethnologue [1] and the Global Mapping Institute [33] (see Text S5, containing analyses that demonstrate representativeness of the sample). Although WALS includes over 2,000 languages, most languages are only defined on a small number of linguistic features.

Table 1 shows the results of three models used to explore the relationships between typological features, and measures of population, geographic spread, and degree of linguistic contact. Population, and to a lesser extent area and number of neighboring neighbors, was a significant predictor for 26/28 of the WALS features that were most relevant to inflectional morphology. Of these, 23 remained significant when language family was partialed out. For 22/28 the demographic variables (population, area over which a language is spoken, and degree of linguistic contact) combined with geographic covariates (latitude/longitude) proved to be better predictors of the linguistic features than geographic location alone. Across a wide range of linguistic features, a systematic relationship (discussed below) between demographic and typological variables was found, providing overwhelming evidence against the null hypothesis that language structure is unrelated to socio-demographic factors. Although the three demographic predictors are not independent (intercorrelations range from .5 to .6), including all three predictors helps to ensure that linguistic-demographic relationships are not spurious. We summarize the findings below (parentheticals refer to entries in Table 1). Text S6 includes more detailed descriptions of the linguistic features.

**Table 1:** Model fits are the Aikake information criteria of models predicting the linguistic feature from just the language family, from population alone, and from the three demographic variables, respectively.

		Model			
Feature	Observed Pattern	Population (Log Speakers)	Area (Log km^2^)	Ling Contact (Log ling. neighbors)	Model Fits
**Morphological Type**					
1. Fusion of inflectional formatives (20) §	Isolating > Concatenating	**$	x	.	358/138/140
2. Inflectional Morphology(26) §	Little or None > Present	**	.	.	688/678/680
**Cases**					
3. *Number of Cases* (49) § (see Figure S1)	Fewer Cases > More Cases	**$	x	x	795/920/912
4. Case Syncretism (28) §	Core/Non-Core Cases > Core Only = No Syncretism	◊$	◊	◊	103/89/93
5. Alignment of Case markings of Full NPs (98) §	Nom/Acc > Erg/Abs	**$	**	**	437/348/349
**Verb Morphology**					
6. Inflectional Synthesis of the Verb (categories per word)(22) § (See Figures 4–5; S1)	Few Forms > Many Forms	**$	**	**	450/451/454
7. Alignment of Verbal Person Marking (100) §	Neutral ≥ Ergative = Accusative > Context Dependent	**	**	x	1083/818/821
**Agreement**					
8. Person Marking on Verbs (102)	None = Agent > Agent & Patient = Patient Only > Agent or Patient	**	**	**	1373/911/923
9. Person Marking on Adpositions (48) §(see Figure 2A)	None > Pronoun > Pronoun + Noun	**$	◊^1^	**	640/498/495
10. Syncretism in Verbal Person/Number Marking (29)	Syncretic > None	**$	◊	**	207/184/188
**Possibility and Evidentials**					
11. Situational Possibility (74) §	Verbal > Morphological	**$	**	**	250/246/249
12. Epistemic Possibility (75) §	Verbal > Morphological	**$	**	**	177/112/112
13. Overlap b/w Epistemic and Situational Possibility (76) §	Situational/Epistemic Collapsed > Separate Markers	**$	◊	**	501/350/350
14. Coding of Evidentiality (77)	No Gram. Evidentials > Gram. Evidentials	**$	.	.	497/536/537
**Negation, Plurality, Interrogatives**					
15. Coding of Negation (112) §	Word > Affix ≥ Double Neg ≥ Particle ≥ Aux. Verb ≥ Word/Affix Variation	**$	**	**	2961/2454/2468
16. Coding/Occurrence of Plurality (34) §	Obligatory > Optional [word > affix/clitic] > None	**$	◊	◊	1055/807/816
17. Associative Plural (36) §	No assoc. Plural > Assoc. Plural	◊$	.	.	200/201/205
18. Polar Question coding (92) §	Question particle > No Question particle	**$	**	**	1022/979/979
**Tense, Possession, Aspect, Mood**					
19. Future Tense (67) § (see Figure 2B)	No Morph > Morph.	**$	◊	◊	320/295/294
20. Past Tense (66) §	Simple Past > No Morph Past > 2–3 Remoteness Dist. > 3+ Remoteness Dist.	**$	◊^1^	◊	617/466/458
21. Perfective/Imperfective (65)	Morph. Distinction > No Morph Distinction	◊$	◊	.	330/303/304
22. Morphological Imperative (70)	Sing only > Not Morph. Marked ≥Sing & Plural ≥ Sing. Syncretic with Plural	**$	x	x	1395/1228/1223
23. Coding of Possessives (57) § (see Figure 2C)	No possessive affix > Possessive Affix	**$	**	**	757/826/828
24. Possessive Classification (59)§	No classification > 2 Classes > 3–5 Classes	**$	**	**	514/477/480
25. Optative (73) §	Not Marked > Morphologically Marked	.	**^1^	x	264/264/250
**Articles, Demonstratives, Pronouns**					
26. Definite/Indefinite Articles (38–39) §	None ≥ Both (Lexical) = Only Def. or Only Indef. ≥ Both (Affixes)	.	**^1^	.	1359/1178/1169
27. Distance distinctions in demonstratives (41)	No distance contrasts > 2 Contrasts ≥ 2+ Contrasts	**$	.	**	501/471/474
28. Expression of Pronominal Subjects (101) §	Oblig. Lexical = Opt. Lexical > Affixes/Clitics	**	◊	**	1102/1011/1012

Smaller values indicate better fits.

§ = Demographics and geographic location predict typology better than geographic location alone (χ2 model comparison (p<.05).

$ = Predictive power of population is reduced (significantly larger residual deviations) by randomly shuffling languages within their families. Indicates that reported effects generalize to within language families.

** = Reported pattern is significant (p between 0.05 and 10^–11^) after controlling for language family.

◊ = Pattern no longer significant (p≥.05) after controlling for language family.

^1^ = Area and Number of Neighbors are significant predictors controlling for population.

. = Consistent with the pattern reported, but not significant.

x = Pattern after controlling for geographic covariates is non-significantly inconsistent with the pattern observed without controlling for geographic location.

Compared to languages spoken in the *esoteric niche* (smaller population, smaller area, fewer linguistic neighbors), languages spoken in the *exoteric niche*:

Are more likely to be classified by typologists as *isolating* languages—those in which grammatical functions are fulfilled by markers not bound to the stem (e.g., modals, lexical items, or particles) than *fusional* languages—those in which grammatical markers show a greater degree of fusion to the stem (e.g., affixes and clitics) (1–2).
Contain fewer case markings (3), and have case systems with higher degree of case syncretism (4) (further reducing the number of morphological distinctions). Nominative/accusative alignment is more prevalent than ergative/absolutive alignment (5).
Have fewer grammatical categories marked on the verb (6) and are less likely to have idiosyncratic verbal morphology such as verbal person markings that alternate between marking agent or patient depending on semantic context (7).
Are more likely to not possess noun/verb agreement or have agreement limited to agents (8) and are more likely to possess no person markings on adpositions (9). As with case markings, syncretism in noun/verb/adposition agreement is more common in languages spoken in the exoteric niche (10).
Are more likely to make possibility and evidentiality distinctions using lexical (e.g., verbal) constructions rather than using inflections such as affixes (11,12,14) and are more likely to conflate the two (semantically distinct) types of possibility (13).
(a) Are more likely to encode negation using analytical strategies (negative word) than using inflections (affixes) and are less likely to have idiosyncratic variations between word and affixation strategies (15). (b) Are more likely to have obligatory plural markers (16). For languages with optional markers, analytic (lexical) strategies are more common than inflectional strategies (affixes or clitics). (c) Are less likely to have a separate associative plural (e.g., “He and his friends”) (17) (c) Are more likely to have a dedicated question particle (18).
(a) Are less likely to encode the future tense morphologically (19) or possess remoteness distinctions in the past tense (20). Languages spoken in the exoteric niche are somewhat more likely to mark the perfective/imperfective distinction in their morphology (21), although this relationship disappears when language geography is partialed out. (b) Are more likely to mark singular imperatives on verbs using inflections than have no morphological markings for imperatives at all, but are less likely to contain more elaborate markings that differentiate between singular and plural imperatives (22). (c) Are less likely to have inflections that mark possession (23). If possession is marked, it is less likely to distinguish between types of possession (e.g., alienable versus inalienable) (24). (d) Are less likely to morphologically mark the optative mood (25).
Are less likely to have definite and indefinite articles (26). If both are present, they are more likely to be expressed by separate words than affixes.
Are less likely to communicate distance distinctions in demonstratives (27).
Are more likely to express pronominal subjects lexically than morphologically (28).

**Figure 2:**
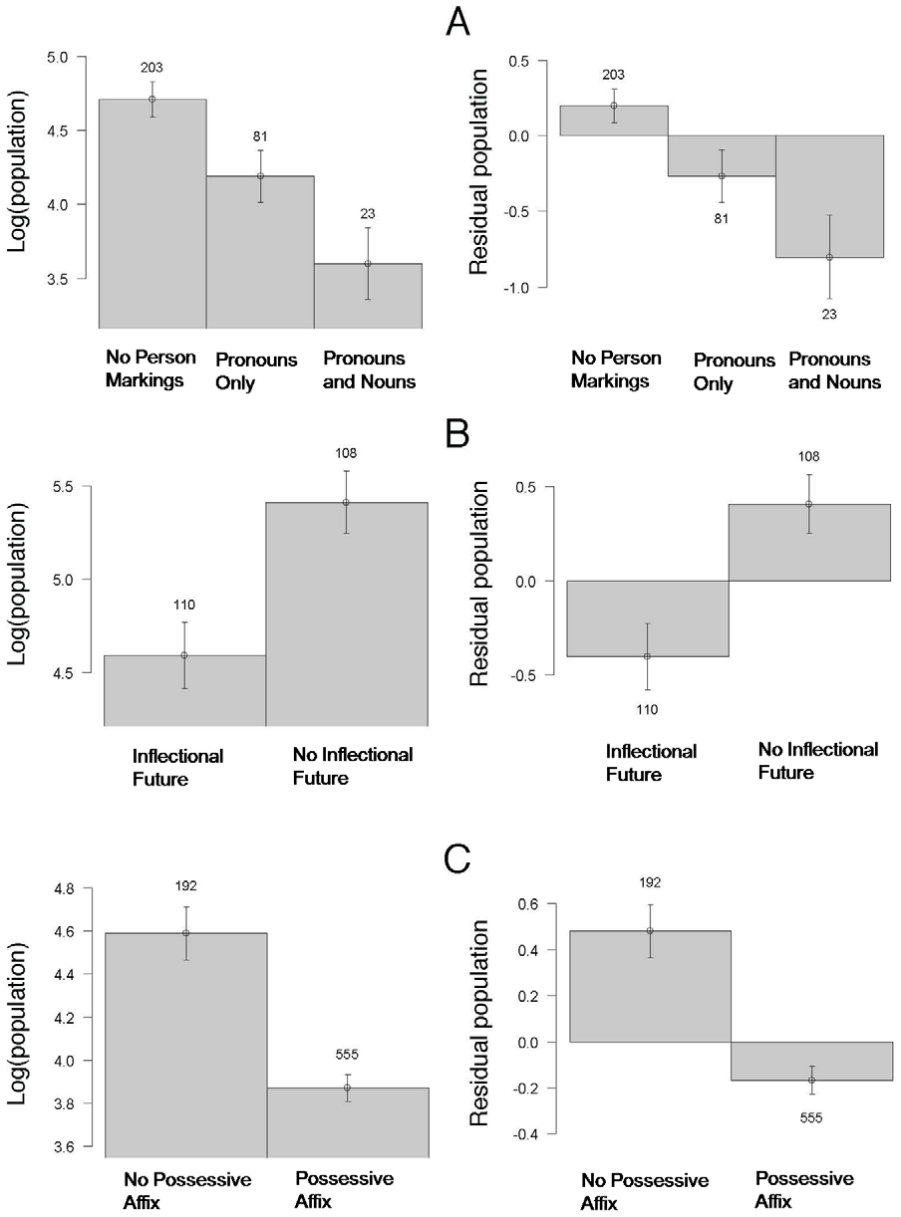
Three features demonstrating the relationship between population and morphological encoding. Y-axis of right-side panels displays residual population after the GLM model partialed out geographic information (reducing the correlation between population and geography to 0). Values above bars represent the number of languages coded for that feature value. (A) Adpositions (prepositions or postpositions) may be coded for person agreement in some languages. In English, there is no such agreement/person marking. One may say “from him” without, for example, encoding onto “from” the gender or number identity of “him,” as opposed to “me” in “from me.” Languages that do encode more information on adpositions show smaller populations. (B) Languages that use inflections (i.e., morphology) for the future tense have smaller populations. (C) Morphological encoding of possession is associated with smaller populations of speakers.

Figure 2 displays features 9, 19, and 23 from Table 1. For each, languages with greater populations are more likely to use a less morphologically complex strategy. Figure S1 shows the relationship between population and two quantitative measures of morphological complexity: number of case markings (feature 3), and inflectional synthesis of the verb (feature 6). Both relationships are significant as analyzed by a GLM : cases, P = .018; inflectional synthesis of the verb: P<.00005 (the relationship remains significant when no-case languages are removed: P = .04) (Figure S1).

**Figure 3:**
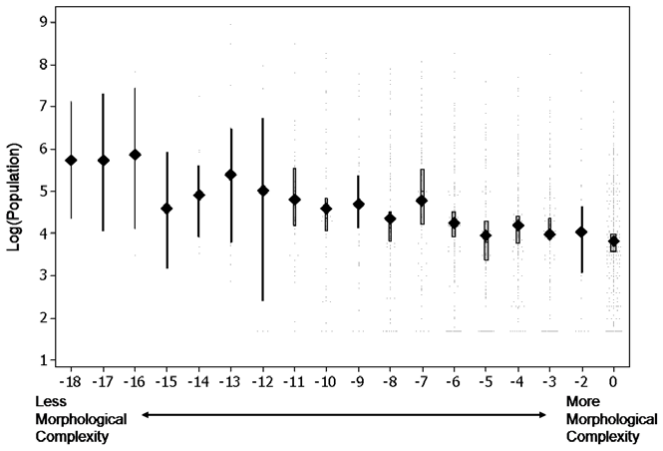
Languages spoken by more people have simpler inflectional morphology. X-axis scores represent a measure of lexical devices compared to the use of inflectional morphology. Filled symbols represent population means for languages with a given complexity score; bars show 95% confidence intervals of the median. Bar width is proportional to sample size for each score.

We constructed a morphological complexity measure by summing the number of features for which each language relies on lexical versus morphological coding and subtracting the total from 0. There was a strong relationship between complexity and speaker population, *p*<.00005 (Figure 3). Languages with the most speakers were more likely to be less morphologically rich, using lexical over morphological strategies for encoding semantic and syntactic distinctions.

**Figure 4:**
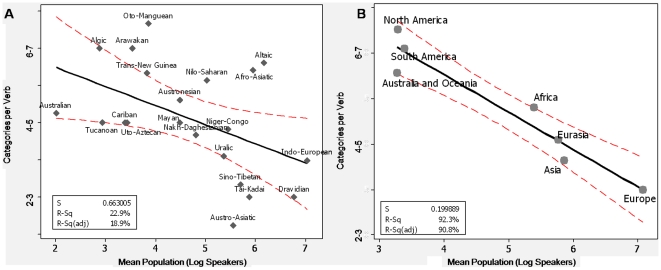
Complexity of verb morphology by language family and geographic regions. (A) Inflectional synthesis of the verb (feature 6 in Table 1) plotted against the mean number of speakers for the largest language families (those containing ≥32 languages). (B) Inflectional synthesis of the verb collapsed by continent. Each point plots the average feature value for the language family. The regression line is flanked by 95% CIs. Eurasia corresponds to the region 38°N–71°N/29°E–172°W.

**Figure 5:**
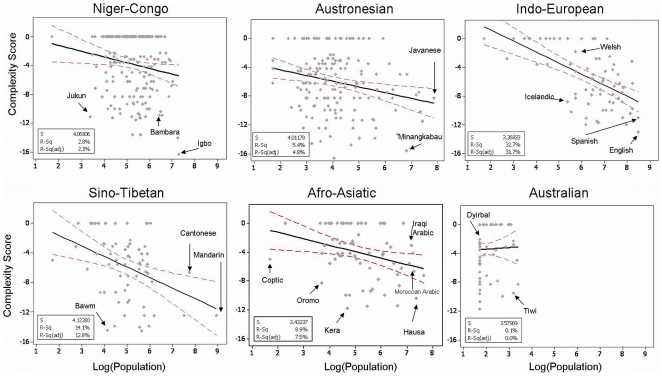
The relationship between population and morphological complexity for the 6 largest language families in our sample. Interestingly, a number of the languages that lie far below the regression line are lingua francas, e.g., Hausa, Bambara, and Oromo are all used as lingua francas (vehicular languages). The Padang dialect of Minangkabau (the second simplest Austronesian language by our measure) is also a lingua franca around West Sumatra, Indonesia.

In cross-cultural or -linguistic research, it is important to consider the issue of non-independence of cases, often subject to autocorrelation (also known as Galton's problem). We controlled for non-independence in several ways:

1. We factored in both language family and geographic location to ensure they did not completely account for the observed language feature distribution (e.g., Figure 2, right panels). Thus, although most linguistic features are subject to strong areal effects, these effects cannot explain the observed findings. Taking as an example one feature (inflectional synthesis of the verb, feature 6), Figure 4 shows the results averaged by the largest language families (Figure 4a, Pearson *r* = .48) and by continents (Figure 4b, Pearson *r* = .96). Figure 5 shows the within-family data for the 6 largest language families in our sample. The relationship with population was significant for each major family (excepting the Australian family which has a very small population range) (see supplementary materials and methods).
2. We also performed a Monte Carlo simulation, randomizing language-demographic information *within* language family. As shown in Table 1 (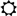 symbols), randomizing within-language family significantly reduces the predictive power of population for 22/28 features.

These controls ensure that the present results cannot be explained as consequences of historical events such as the colonization of the New World (and the population reduction that ensued) [34].

## Discussion

Languages that are on the exoteric side of esoteric-exoteric continuum—as indicated by larger speaker populations, greater geographical coverage, and greater degree of contact with other languages—had overall simpler morphological systems, more frequently express semantic distinctions using lexical means, and were overall less grammatically specified. This was true both for quantitative grammatical measures such as the number of different grammatical categories encoded by verbal inflections (feature 6) and case markings, as well as for qualitative grammatical types. For example, languages spoken in the exoteric niche were associated with a lack of conventional strategies for encoding semantic distinctions like situational/epistemic possibility, evidentiality, the optative, indefiniteness, the future tense, and both distance contrasts in demonstratives (consider the rarity of the English “over yonder”) and remoteness distinctions in the past tense.

With few exceptions, the same patterns were observed whether population, area, or linguistic contact was used in the model. Overall, the population model provided the greatest predictive power.

As noted above, semantic distinctions coded lexically are more likely to be optionally expressed than those coded inflectionally (e.g., lexical versus inflectional encoding of tense). Thus, languages that are less grammatically specified tend to rely more on extra-linguistic information such as pragmatics and context [13]. Reduced reliance on morphology also has the effect of increasing the transparency between word-forms and meanings (form-meaning compositionality) [2]. Consider the high occurrence of exceptions in the inflectionally marked past tense forms of English compared to the perfect regularity of the modally marked future tense. One reason for the inverse relationship between morphology and form-meaning compositionality is that inflections such as affixes are, by definition, phonologically bound to the stem, which increases opportunities for phonological compression and sound change to disrupt regular mappings between form and meaning. Thus, although it is logically possible to have complex inflectional morphology that is highly regular (frequently classified as agglutination), in practice, coarticulation, historical sound change, and other phonological/articulatory processes often subvert this regularity and lead to more idiosyncratic mappings [35]–[37]. We found that the relationship between exotericity and increased form-meaning compositionality holds not only for specific linguistic features like tense and evidentiality, but is also supported by the observation that languages in the exoteric niche are more likely to be classified by typologists as being isolating rather than concatenative or fusional [38].

### The Linguistic Niche Hypothesis

Our results provide strong evidence for a relationship between social structure and linguistic structure. Here, we speculate about the social and cognitive mechanisms that may give rise to this relationship. The linguistic niche hypothesis (LNH) provides one framework in which to consider two central questions raised by the present analyses: (1) Why are languages spoken in the exoteric niche morphologically simpler than languages spoken in the esoteric niche? (2) Why are languages spoken in the esoteric niche so morphologically complex, given that such a high level of specification seems unnecessary for communication?

We tentatively propose that the level of morphological specification is a product of languages adapting to the learning constraints and the unique communicative needs of the speaker population. Complex morphological paradigms appear to present particular learning challenges for adult learners even when their native languages make use of similar paradigms [39]. As a language spreads over a larger area (e.g., as a result of colonization) and is being learned by a greater number of adult learners, complex morphological paradigms have a greater probability, over historical time, to become simplified [28], [26], [12]. This appeal to learning constraints of adult learners as an explanation for morphological simplification has also been proposed by the descriptive analyses of Trudgill [29] and McWhorter's (“interrupted transmission” hypothesis) [7] which has been previously supported only by selected examples. Morphological simplification following spread may greatly reduced through prescriptivism (namely, formal instruction) as was common in the case of the spread of Russian in the 20^th^ century.

With increased geographic spread and an increasing speaker population, a language is more likely to be subjected to learnability biases and limitations of adult learners (Text S7). Linguistic change that facilitates adult second-language learning will accumulate over historical time (calculating that rate of change is an intriguing topic that is beyond the scope of the present work). It appears that morphological simplification (and frequently accompanying increases in the transparency of form-to-meaning mapping [2]) comprises a major type of such change (see SI for additional analyses). It is important to note that adult learners can affect the trajectory of a grammar even when they make up a minority of the population (Text S8).

The *LNH* offers a functionalist account of why morphological paradigms often extend far beyond communicative necessity. Despite well-specified theories of both the synchronic and diachronic processes of grammaticalization that describe the steps that lead to increases in morphology [36], [40], [41], the morphological overspecification so common to languages has remained a puzzle: *Why* are some languages so much more grammatically specified than others? (21, 38) We propose that the surface complexity of languages arose as an adaptation to the esoteric niche and is the result of a pressure to facilitate learning of the language by infants (without regard for adult learnability which is irrelevant for languages that are not being learned by adults). As noted above morphologically overspecification correlates with redundancy (Text S9). What appears to be functionless overspecification may provide infants with multiple cues allowing language acquisition to proceed with less reliance on extralinguistic context. Communication is typically linguistically underspecified; adults may cope with such underspecification more effectively than infants and thus it is infants that would benefit most from linguistic redundancy [43]–[45].

In Text S10 we formalize this intuition as a mathematical model and derive a linguistic learnability (fitness) landscape for languages over varying levels of morphological specification and proportions of adult learners. Making several basic assumptions, we show that as the proportion of L2 learners increases, greatest language fitness is obtained for languages that minimize grammatical distinctions while decreasing redundancy (Text S10); as the proportion of L1 increases, language structure is increasingly determined by redundancy increasing the likelihood of languages with more complex morphological systems. To test a critical assumption of the model—redundancy is greatest for languages with few L2 learners—we compared a translation of a document into 103 languages and observed a highly significant correlation (*r* = −.56, *P*<.0005) between population and redundancy (see Text S11). This result, which was obtained without any explicit coding of linguistic features, also serves as an independent confirmation of the main finding obtained in the main analysis: languages with more speakers are less morphologically specified than languages with fewer speakers.

The paradoxical prediction that morphological overspecification, while clearly difficult for adults, facilitates infant language acquisition is novel and is empirically testable. In Text S12 we present some support for the more general prediction that the most frequent typologies (e.g., case suffixes are much more widespread/frequent than case prefixes) correspond to those known to be more easily learned by children whereas typologies common to high-population (i.e., exoteric) languages are those that are best learned by adults (see also Figure S2; Table S2). Direct empirical testing contrasting adult versus child learning of different linguistic features is clearly required.

The linguistic niche hypothesis stresses redundancy as the force that results in greater inflection in languages with few speakers. An alternative is that languages with fewer speakers may come to rely more on inflectional rather than lexical devices because these afford greater economy of expression. On average a language with a greater reliance on inflectional devices will produce shorter sentences than one that relies on lexical devices [46]. Assuming that economy of expression is constrained by what can be learned as well as a pressure for languages to be clear [e.g., 47], this account still predicts that morphological complexity will vary as a function of the learning population. That is, morphological paradigms, while potentially allowing for greater economy of expression, are more difficult to learn (and perhaps comprehend) by adults, and so will tend to be avoided in languages with many adult learners. Another possibility is that complex morphology in languages with few speakers was not selected for any functional reason, but is the product of drift combined with faithful transmission in a small speaker population. On this account, larger populations can buffer against fixation of nonfunctional or deleterious variants [e.g.], [48,49]. One way to discriminate between these alternatives is through a systematic comparison of the learnability of various grammatical devices by children and adults (see Text S12).

We have presented statistical evidence showing that aspects of morphological structure are predicted from nonlinguistic demographic variables, especially population. These results provide support for a non-arbitrary relationships between linguistic and social structure. One way to understand how these relationships come about is through what we have referred to as the Linguistic Niche Hypothesis (LNH) according to which different languages are placed under different learning constraints by socio-demographic factors. Languages spoken by millions of people over a diverse region are under a greater pressure to be learnable by adult outsiders. This pressure gradually results in morphological simplification with an increase in productivity of existing grammatical patterns, and greater analytical and compositional structure [2]. A language spoken by relatively few people over a small area is less subject to these same pressures. Idiomatic constructions and “baroque accretion” so common to languages is more likely to flourish in an environment composed exclusively of young native learners. Such constructions increase encoding redundancy which may aid acquisition by first language learners whose learning systems are more capable of handling increased morphosyntactic complexity. We view the LNH as an initial step in understanding the mechanisms by which social structure affects grammatical structure and readily acknowledge the usefulness of case-studies (both linguistic and cultural) that are the norm in anthropology, and descriptive linguistics [text S3; 50]. In combination with these prior case studies, the associations reported in the present work offer a glimpse into potentially far richer relationships that may exist between grammar and culture. Analyses of data from cross-cultural repositories, e.g., SCCS [51], and the use of phylogenetic/biological cladistic methods [52], [53], promise to provide additional insights into the relationships between social and linguistic structure.

## Materials and Methods

We used three socio-demographic variables as proxies for esotericity: speaker population, geographic spread, and degree of inter-language contact. Speaker population data for each language was retrieved from the Ethnologue [1] and included the summed total of speakers in all the countries in which the language is spoken. Total area (km^2^) for each language was calculated from data provided by Global Mapping International [33]. Inter-linguistic contact was calculated based on languages boundaries: for each language we counted the number of languages contained in, overlapping with, or contacting the area polygons of other languages. Linguistic data was retrieved from WALS [32]. We selected linguistic features most relevant to inflectional morphology. Details are presented below.

### Geographic/Demographic Variables

Because direct measures for the esotericity are not available on a large scale, we used three proxy variables: speaker population, geographic spread, and degree of inter-language contact. Speaker population data for each language was retrieved from the Ethnologue [1] and included the summed total of speakers in all the countries in which the language is spoken. Because nonnative speaker population estimates are unreliable and unavailable for most languages in our sample, our population estimates were conservative, including only native speakers, as reported by the Ethnologue. Populations of less than 50 speakers were set to 50. Area (km^2^) for each language was calculated from data provided by Global Mapping International [33]. These data contained boundary information (global mapping polygons) for most of the languages in WALS. The area measure was the sum of all the geographic regions in which the language is spoken. Inter-linguistic contact was calculated based on languages boundaries: for each language we counted the number of languages contained in, overlapping with, or contacting the area polygons of other languages. For example, although English originates in the British Isles, the fact that it is spoken in North America and Australia means that its neighbors include the extant indigenous languages of those continents.

### Selecting Typological Features for Analysis

Our analyses focused on typological factors most relevant to morphological encoding with particular emphasis on continuous variables such as the number of inflectional case markings or the inflectional synthesis of verbs—the number of different types of information that can be inflectionally encoded by verbal affixes—measured in categories per word [54]. An additional guide for feature selection was the ability to make *a priori* predictions about the level of morphological complexity of a given feature. For instance, plurality (feature 16) can be coded using prefixes, suffixes, some combination of the two, a plural word, a plural clitic, reduplication, or by using non-conventionalized lexical means. Clearly, languages that have morphological coding of plurality are more grammatically specified in this respect than languages that do not. We made no *a priori* predictions about the relative morphological complexity of prefixes versus suffixes versus reduplication. However, our analyses revealed that demographic factors in fact correlated strongly with prefixing versus suffixing strategies in a range of linguistic domains and we include these additional analyses below.

Although our corpus included 2,236 languages, no feature was defined for all the languages in the WALS database. The results presented in Table 1 are based on a median of 218 languages per feature analyzed (range: 112–1,074). The data in WALS are limited to existing linguistic descriptions. In subsequent analyses we show that WALS representatively samples the world's languages.

### Notes on Statistical Analyses

Typological variables with no natural ordering were predicted using multinomial regression (proportional odds logistic regression). Binary variables were predicted using simple logistic regression (logit GLM), continuous variables (features 3, 6, 24, 27) were predicted using a Gaussian GLM. The included analyses partial out language location by including as covariates the latitude/longitude coordinates of the language as reported in WALS. We also ran analyses that partialed out location by including the continent as a random effect. These analyses resulted in larger uncertainties in the typological value estimates, but in no case led to discrepant conclusions.

Because many languages only had information for a few of the features listed in Table 1, we divided the overall morphological complexity score (plotted in Figures 3 and 5) by the proportion of the features present, effectively controlling for the sparseness of the data. Languages had to be defined on at least 3 features from Table 1 to be included in the analysis. The scores used in Figures 2 and 5 plot the adjusted complexity scores; in Figure 2 they were rounded to the nearest integer for graphing purposes. The 0 values in Figures 3 and 5 correspond mostly to languages with very sparse linguistic data available in WALS. Their removal does not qualitatively affect the analysis.

## Supporting Information

Figure S1 The relationship between population and number of nominal cases (a), and number of categories per verb (b). The regression lines are flanked by 95% CIs. The ranges on the x-axis correspond to the coding of these features in the World Atlas of Langauge Structures.
(0.10 MB DOC)

Figure S2 Word order and affixation frequencies and associated speaker populations. a. Distribution of word order types versus the mean speaker populations (numbers above bars indicate number of languages with the given feature value). b. Speaker population adjusted by geography. c–d. A break-down of languages classified as having dominantly prefixing versus dominantly suffixing inflectional morphology.
(0.10 MB DOC)

Table S1 Examples of native (L1) to non-native (L2) populations for several languages.
(0.03 MB DOC)

Table S2 A comparison of linguistic features (typologies) that are most common to languages in the exoteric niche compared to overall typological frequency.
(0.05 MB DOC)

Text S1 A note regarding Japanese as an example.
(0.02 MB DOC)

Text S2 A note about the correlations between our main demographic variables.
(0.02 MB DOC)

Text S3 A note regarding our nomothetic approach.
(0.02 MB DOC)

Text S5 A representative analysis of our language sample.
(0.35 MB DOC)

Text S4 A note about esoteric and exoteric uses of a language.
(0.02 MB DOC)

Text S6 A detailed description of the linguistic features used.
(0.06 MB DOC)

Text S7 A note regarding multilingualism.
(0.02 MB DOC)

Text S8 A note regarding generational transmission of a nonnative language.
(0.03 MB DOC)

Text S9 A clarification of the term “redundancy.”
(0.02 MB DOC)

Text S10 Modeling language fitness as a function of age of acquisition.
(0.56 MB DOC)

Text S11 Text compressibility as a measure of linguistic redundancy.
(0.10 MB DOC)

Text S12 Supporting analyses of constituent order and application to adult versus child language acquisition.
(0.04 MB DOC)
