# Supplementary material for: Language Structure Is Partly Determined by Social Structure
Source: PLoS One. 2010 Jan 20;5(1):e8559. doi: 10.1371/journal.pone.0008559 (PMC2798932; doi:10.1371/journal.pone.0008559)

### Figure S1. The relationship between population and number of nominal cases (a), and number of categories per verb (b). The regression lines are flanked by 95% CIs. The ranges on the x-axis correspond to the coding of these features in the World Atlas of Langauge Structures


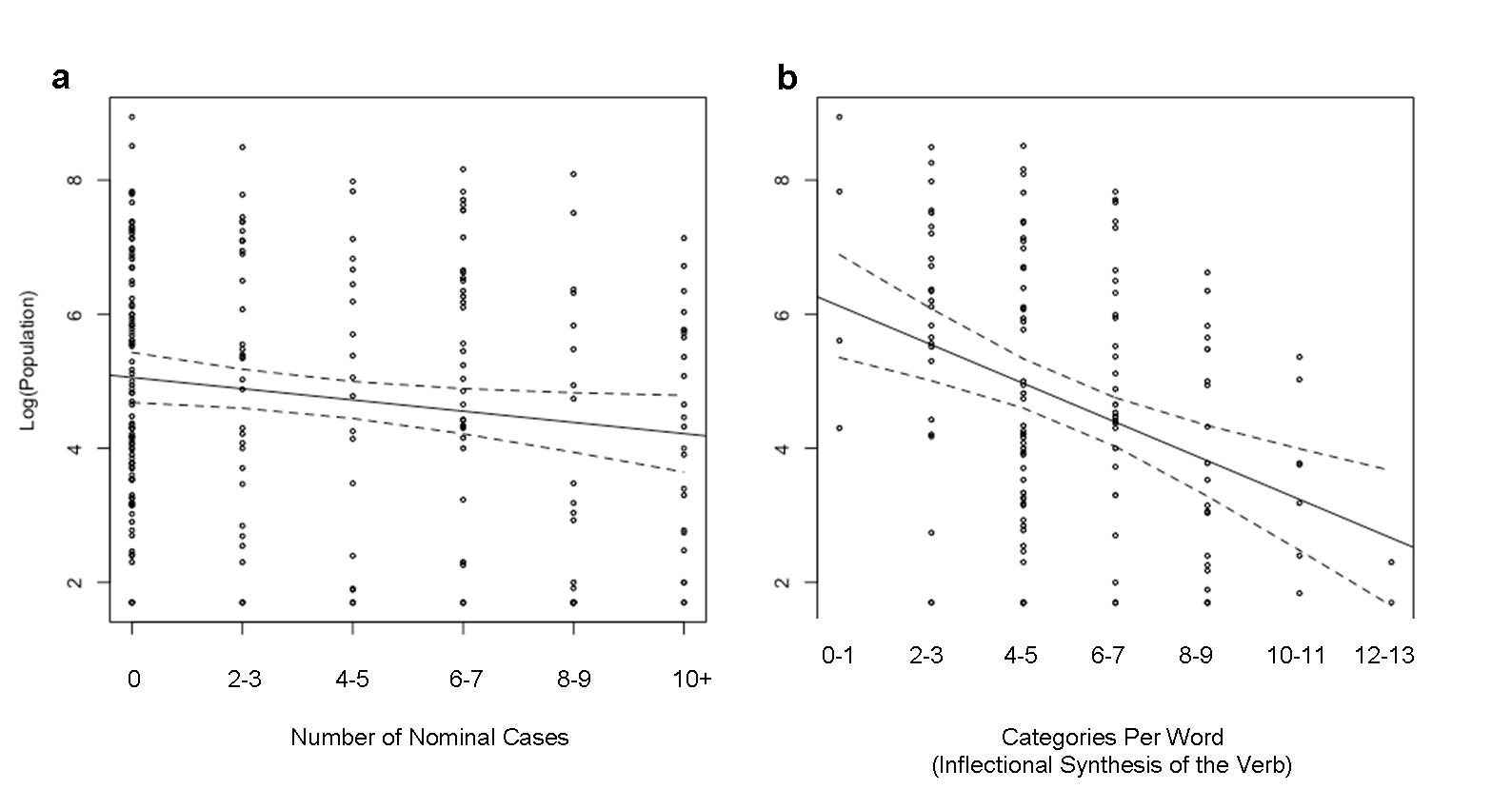

Supplement: Figure S1 — The relationship between population and number of nominal cases (a), and number of categories per verb (b). The regression lines are flanked by 95% CIs. The ranges on the x-axis correspond to the coding of these features in the World Atlas of Langauge Structures. (0.10 MB DOC) [file pone.0008559.s001.doc]
