# Supplementary material for: Language Structure Is Partly Determined by Social Structure
Source: PLoS One. 2010 Jan 20;5(1):e8559. doi: 10.1371/journal.pone.0008559 (PMC2798932; doi:10.1371/journal.pone.0008559)

Figure S2. Word order and affixation frequencies and associated speaker populations. a. Distribution of word order types versus the mean speaker populations (numbers above bars indicate number of languages with the given feature value). b. Speaker population adjusted by geography. c-d. A break-down of languages classified as having dominantly prefixing versus dominantly suffixing inflectional morphology.

**a. b.**


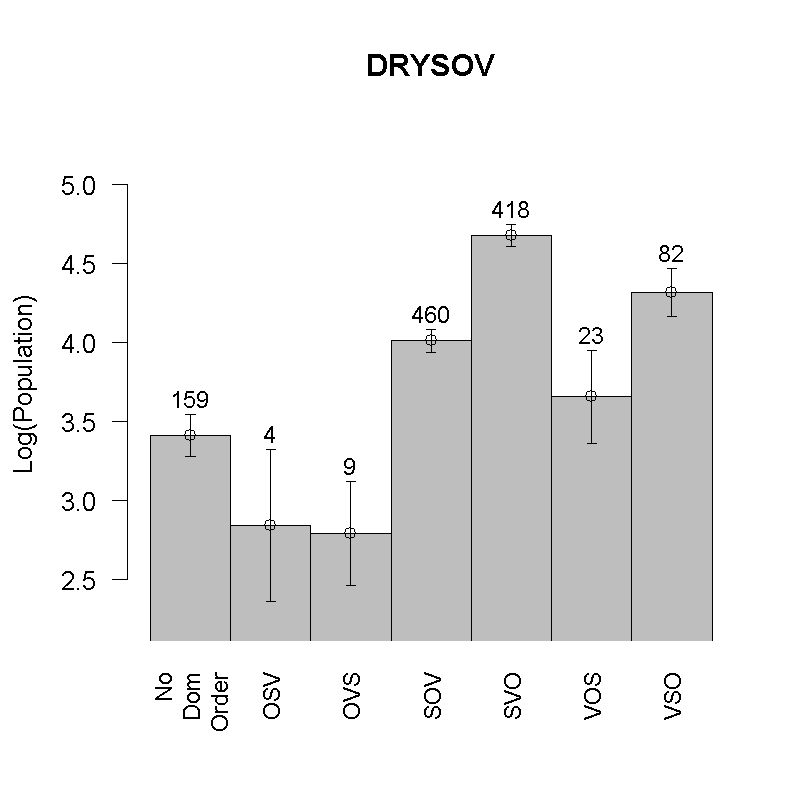

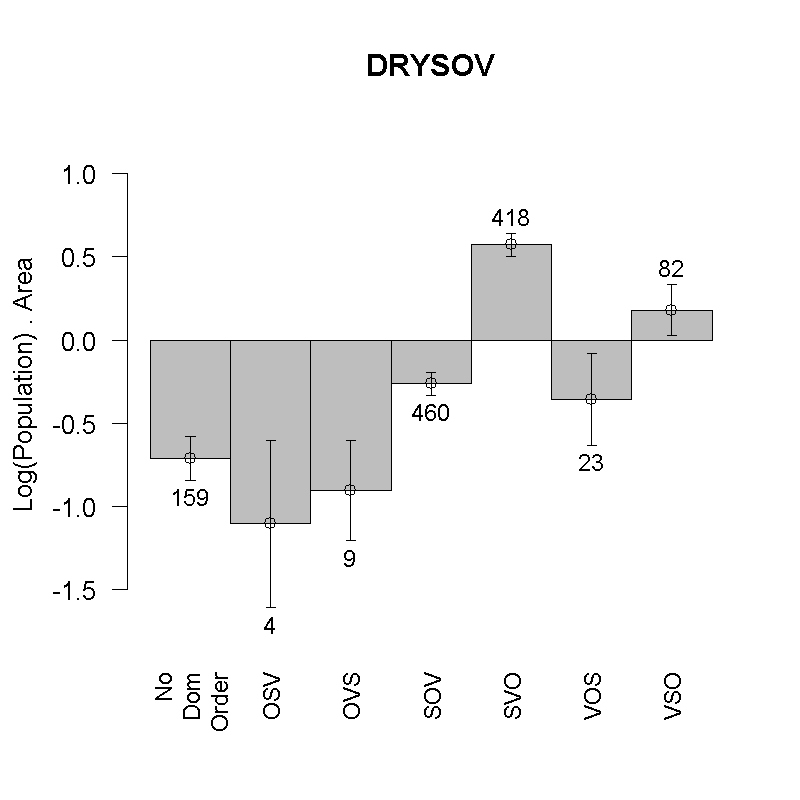


**c. d.**


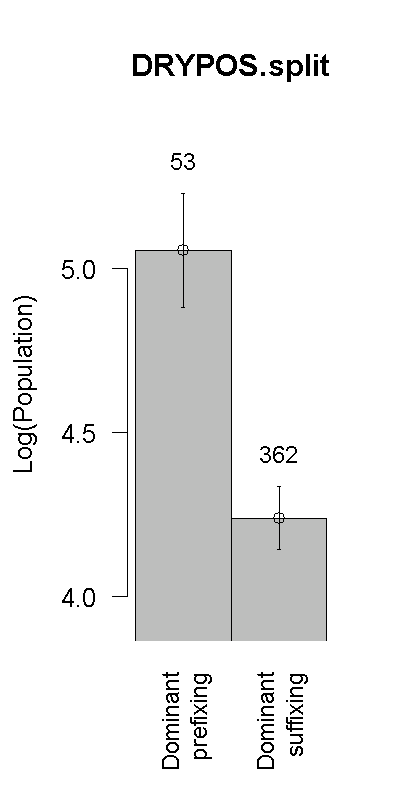

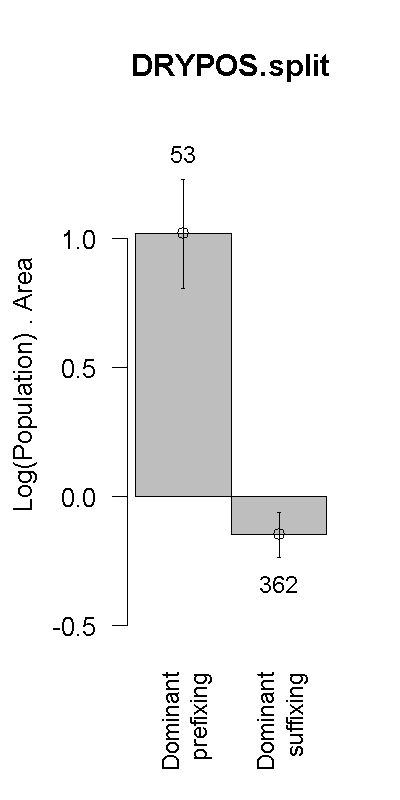

Supplement: Figure S2 — Word order and affixation frequencies and associated speaker populations. a. Distribution of word order types versus the mean speaker populations (numbers above bars indicate number of languages with the given feature value). b. Speaker population adjusted by geography. c–d. A break-down of languages classified as having dominantly prefixing versus dominantly suffixing inflectional morphology. (0.10 MB DOC) [file pone.0008559.s002.doc]
