# Supplementary material for: Language Structure Is Partly Determined by Social Structure
Source: PLoS One. 2010 Jan 20;5(1):e8559. doi: 10.1371/journal.pone.0008559 (PMC2798932; doi:10.1371/journal.pone.0008559)
Supplement: Table S1 — Examples of native (L1) to non-native (L2) populations for several languages. (0.03 MB DOC) [file pone.0008559.s003.doc]

### Table S1: Examples of native (L1) to non-native (L2) populations for several languages.

| **Language** | **Speakers (millions)**[16] | | |
| --- | --- | --- | --- |
|  | L1 | L2 | %L1 |
| Malay | 30 | 170 | .15 |
| English | 330 | 812 | .29 |
| French | 65 | 50 | .57 |
| Amharic | 27 | 7 | .79 |
| Abkhaz | 0.11 | .006 | .95 |
| *Siberian Yupik Eskimo* | 0.001 | ~0 | ~1 |
