# Supplementary material for: Language Structure Is Partly Determined by Social Structure
Source: PLoS One. 2010 Jan 20;5(1):e8559. doi: 10.1371/journal.pone.0008559 (PMC2798932; doi:10.1371/journal.pone.0008559)
Supplement: Table S2 — A comparison of linguistic features (typologies) that are most common to languages in the exoteric niche compared to overall typological frequency. (0.05 MB DOC) [file pone.0008559.s004.doc]

### Table S2: A Comparison of linguistic features (typologies) that are most common to languages in the exoteric niche compared to overall typological frequency.

|  |  | | | | |  |  |
| --- | --- | --- | --- | --- | --- | --- | --- |
| Feature  (WALS Chapter number)  Descriptions available at:  wals.info/feature/<chap#> | Most prevalent typology based on demographics | Population  (Log Speakers) | Area  (Log km^2^) | Ling Contact  (Log ling. neighbors) | Model Fit | Most prevalent typology based on frequency (number of language families with listed pattern:opposite pattern) | |
| 1. Position of inflections (26) § | Prefixes | **☼ | . | ** | 1342 | Suffixes (66:14) | |
| 1. Position of Case Affixes (51) § | Prefixes/Prepositonal Clitics | ** | - | - | 331 | Suffixes/Postpositional Clitics  (126:22) | |
| 1. Type of possessive affix (57) § | Suffix | **☼ | ** | ** | 382 | Prefix (70:45) | |
| 1. Word Order (81) | SVO | ** | - | - | 1161 | SOV (106:41) | |
| 1. Type of Adpositions (85) § | Prepositions | ** | - | - | 854 | Postpositions (115:45) | |
| 1. Noun-Genitive Order (86) § | Noun+Gen | ** | ** | * | 912 | Gen+Noun (120: 36) | |
| 1. Noun-Adjective Order (87) § | Adj+Noun | . ☼ | ** | . | 962 | Noun+Adj (89:63) | |
| 1. Noun-Demonstrative Order (88) § | Noun+Dem | ** | . | ** | 946 | Dem+Noun (97:48) | |
| 1. Noun-Numeral Order (89) § | Noun+Num | ** | . | ** | 820 | Num+Noun (84:59) | |
| 1. Noun-Relative Clause Order (90) § | RelClause+Noun | **☼ | ** | ** | 338 | Noun+RelClause (63:29) | |
| 1. Noun-Degree modifier Order (91) § | Deg+Adj | **☼ | ** | ** | 385 | Deg+Adj (56:35) | |

*Model Fits* are the Aikake Information Criteria of models predicting the linguistic feature from the three demographic variables.

** = pattern is significant (p<.05) after controlling for langauge family

* = pattern no longer significant (p>.05) after controlling for language family

§ = demographics and geographic location predict typlogy better than geographic location alone (Chi-sq model comparison, p<.05)

☼ = Predictive power of *population* is reduced (significantly larger residual deviations) by randomly shuffling languages within their families. Indicates that reported effects generalize to *within* language families.

. = consistent with the pattern reported, but not significant

- = No significant effects
