# Supplementary material for: Language Structure Is Partly Determined by Social Structure
Source: PLoS One. 2010 Jan 20;5(1):e8559. doi: 10.1371/journal.pone.0008559 (PMC2798932; doi:10.1371/journal.pone.0008559)
Supplement: Text S1 — A note regarding Japanese as an example. (0.02 MB DOC) [file pone.0008559.s005.doc]

**Text S1**

For example, Japanese is spoken by ~122 million as a first language and only ~1-2 million as a second language.
