# Supplementary material for: Language Structure Is Partly Determined by Social Structure
Source: PLoS One. 2010 Jan 20;5(1):e8559. doi: 10.1371/journal.pone.0008559 (PMC2798932; doi:10.1371/journal.pone.0008559)
Supplement: Text S2 — A note about the correlations between our main demographic variables. (0.02 MB DOC) [file pone.0008559.s006.doc]

**Text S2**

The Pearson correlation between population and area (km2) in our dataset is 0.55; between population and number of linguistic neighbors, 0.60.
