# Supplementary material for: Language Structure Is Partly Determined by Social Structure
Source: PLoS One. 2010 Jan 20;5(1):e8559. doi: 10.1371/journal.pone.0008559 (PMC2798932; doi:10.1371/journal.pone.0008559)
Supplement: Text S3 — A note regarding our nomothetic approach. (0.02 MB DOC) [file pone.0008559.s007.doc]

**Text S3**

Historically, accounts of relations between language and culture have been idiographic in nature, focusing on the histories of specific languages (12). The present work is nomothetic in its approach. We recognize that there are languages that violate each of the specific claims we make. An understanding of the “exceptions” can only be achieved by augmenting the present approach with idiographic studies of specific languages. The conclusions of the statistical analyses do not depend on the correctness of the theoretical framing, based on work that is considered somewhat controversial (12, 21, 7, 24, 42, 5, 2). We offer the Linguistic Niche Hypothesis as a hypothesis in wait for direct empirical tests.
