# Supplementary material for: Language Structure Is Partly Determined by Social Structure
Source: PLoS One. 2010 Jan 20;5(1):e8559. doi: 10.1371/journal.pone.0008559 (PMC2798932; doi:10.1371/journal.pone.0008559)

### Text S5: Representativeness Analysis

The WALS and Ethnologue databases are the only current sources of data with sufficient coverage of the world’s languages to permit the type of analyses we conduct. Indeed, our full sample contains about a third of the world’s 6000+ languages, limited primarily by the descriptive information being unavailable for the remaining languages. It is important, however, to show that (1) the full sample provides a sufficient coverage of the world’s languages, and (2) that the subset of languages for which the relevant morphological information is available is also representative. This analysis is shown in the figures below. Panel a shows that the WALS sample provides broad coverage and a geographically representative sample of the world’s languages. Panel b shows the distribution for the 6 largest language families (accounting for 68% of all the world’s languages). WALS again provides a relatively representative sample. We also conducted analyses to ensure that the subset of the WALS languages which were defined on the features relevant to the present analyses still provided for wide geographic coverage. The analyses for the two main features—Number of Cases (F3-W49) and Categories per Word (F6-W22)—is shown in Panel c, and again show broad geographic coverage.

Despite the broad coverage of the present sample, there were significant deviations (as measured by χ^2^ tests) between the Ethnologue and the WALS samples, most notably an under-representation of African languages and a relative over-representation of New World languages. Focusing on the 6 largest language families, Niger-Congo languages and Trans New-Guinean languages were under-represented, while Australian language were over-represented. This pattern of over/under-representation can be explained by simply noting that geographic areas and language families with the most number of languages are most likely to be under-represented.

These deviations do not detract from the strength of the present conclusions because, rather than being interested in, e.g., the exact value of the average number of cases that European languages have, we focus on relationships between socio-demographics and linguistic factors, which do not depend on having a sample that exactly matches the full inventory of the world’s languages.

### Comparisons of distributions by broad geographic (a, c) and language-family (b) areas of the WALS sample and the complete list of languages as reported in the Ethnologue.

a.


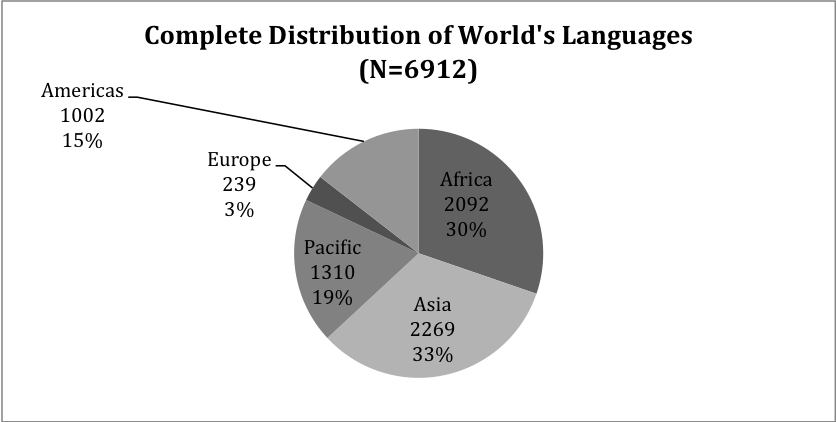

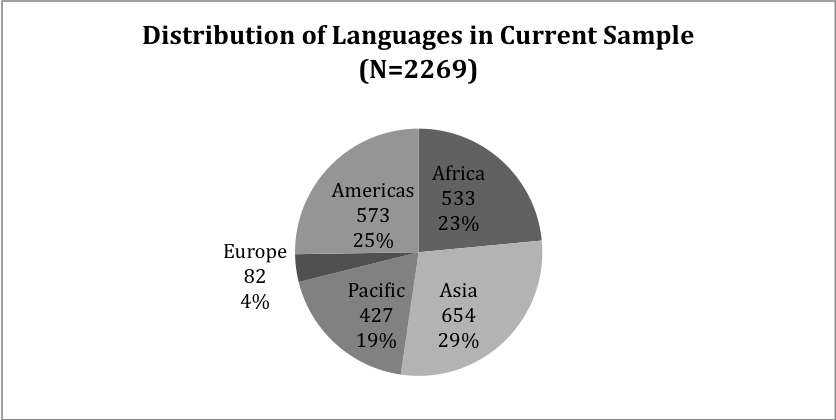


b


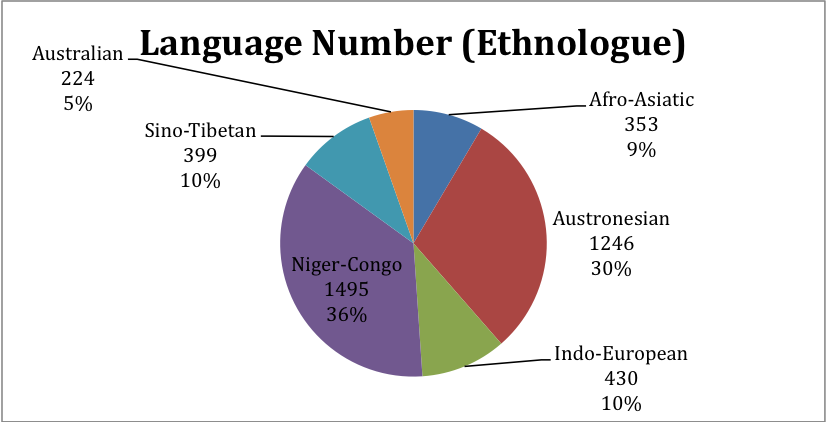

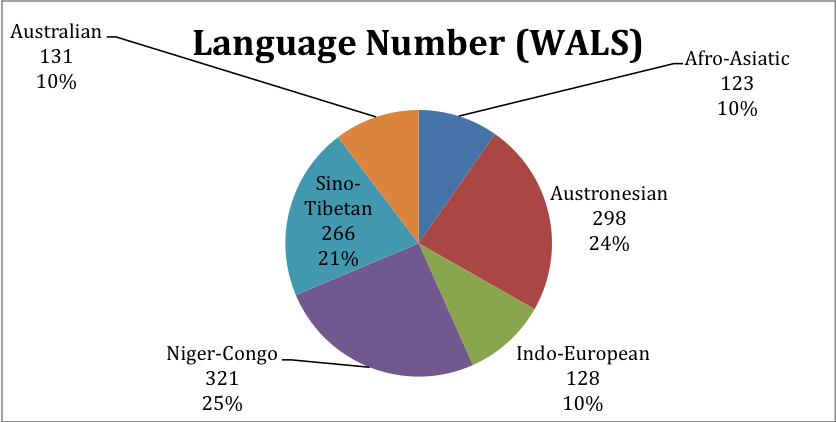


c.


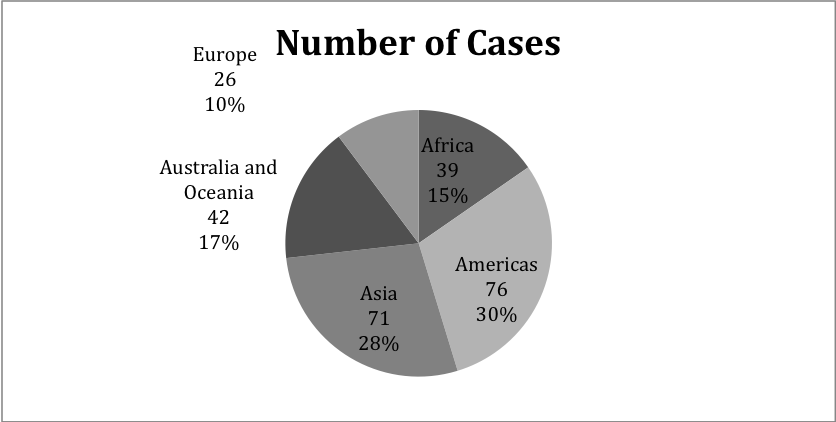

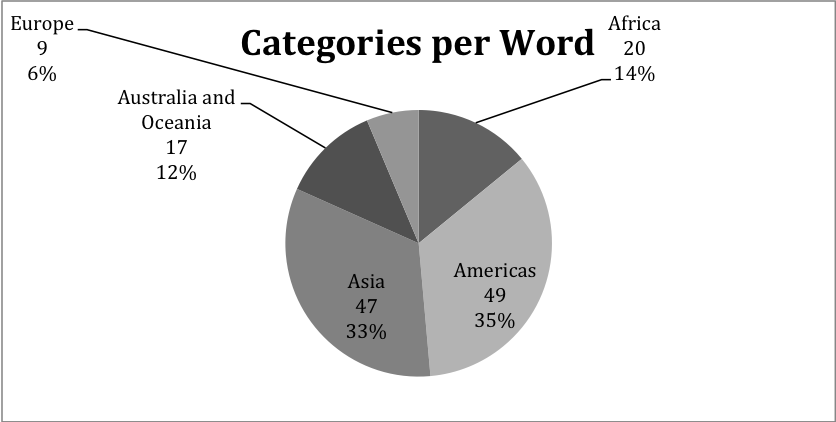

Supplement: Text S5 — A representative analysis of our language sample. (0.35 MB DOC) [file pone.0008559.s008.doc]
