# Supplementary material for: Language Structure Is Partly Determined by Social Structure
Source: PLoS One. 2010 Jan 20;5(1):e8559. doi: 10.1371/journal.pone.0008559 (PMC2798932; doi:10.1371/journal.pone.0008559)
Supplement: Text S4 — A note about esoteric and exoteric uses of a language. (0.02 MB DOC) [file pone.0008559.s009.doc]

**Text S4**

It is more accurate to refer to esoteric and exoteric usages of a language rather than a language being spoken in an esoteric versus exoteric settings; it is typical for languages to exhibit clines within which within-group and between-group communicative functions are fulfilled. For example, Trinidadians commonly speak a colloquial variety of English amongst themselves, but standard English to outsiders. Thus, the linguistic niche refers to a socio-linguistic environment rather than a physical environment.
