# Supplementary material for: Language Structure Is Partly Determined by Social Structure
Source: PLoS One. 2010 Jan 20;5(1):e8559. doi: 10.1371/journal.pone.0008559 (PMC2798932; doi:10.1371/journal.pone.0008559)
Supplement: Text S6 — A detailed description of the linguistic features used. (0.06 MB DOC) [file pone.0008559.s010.doc]

### Text S6: Descriptions of Linguistic Features

The text below briefly summarizes and provides relevant examples for some of the features used in Table 1 of the main text. Examples are taken from the referenced chapters of WALS. Each section is prefixed by a feature code used in Table 1 (e.g., F1) and the WALS chapter number (e.g., W20). Full descriptions of the features can be obtained from the online version of WALS: [http://wals.info/feature/description/#](http://wals.info/feature/description/) where # is the WALS feature number.

| **F3-W49** | **Nominal case markings** are morphological devices that specify the relationship between a noun and its head. Cases markings are often affixes and specify roles such as agency, patient, instrument, possession, and location. An example of a language with a very large case system is Hungarian (20-21 productive cases) and Kayardild—a language of Australia (20 cases). The WALS feature “borderline case markings” was excluded due to idiosyncratic coding. |
| --- | --- |
| **F5-W98** | **Case alignment** refers to the mapping between case markings and syntactic role. In nominative/accusative languages there is an equivalence between the agent of a transitive verb and the core argument of an intransitive verb, which are thus treated alike, and are distinct from the object of a transitive verb. In **ergative/absolutive** languages, there is an equivalence between the object of a transitive verb and the single core argument of an intransitive verb, which contrast with the agent of a transitive verb. |
| **F6-W22** | Grammatical categories like tense, voice, or agreement can be expressed either by individual words (analytically) or by affixes attached to some other word or a word-stem (synthetically). **Inflectional synthesis of the verb** is simply a count of the number of categories per word (cpw) that are marked through such synthetic stems. For example, Vietnamese has no inflectional verbal morphology and so has 0 cpw. English verbs have two forms, inflecting for the past tense and the subject. In contrast verbs in Georgian can inflect for a maximum of 9 categories and in Koasati—an indigenous language in Southern U.S—for 13. |
| **F[4,10]-W[28,29]** | **Syncretism** refers to distinct morphological forms having an identical realization. For example, in English the nominative and accusative forms of the 2^nd^ person pronoun are syncretic (“you”), but remain in the 3^rd^ person (he/him; she/her). |
| **F[8,9]-W[102, 48]** | In many languages verbs and nouns must “agree,” most commonly for person (first, second, third) number, in some languages for gender, but sometimes for gender, animacy, and definiteness. Such **verbal** **person markings** can depend on the agent, the patient, both agent and patient, or either one. In the latter case (e.g., Nocte, a language of India), whether the verb must agree with the agent or patient depends on which is higher in a person hierarchy (e.g., 1^st^ > 2^nd^ > 3^rd^). **Person** **marking on adpositions** code whether adpositions have to agree with person. English adpositions have no person markings: compare, “I sat *across* from him” vs. “He sat *across* from me.” By contrast, in some languages the term for “across” would inflect differently for “him” and “me.” Such agreement can be limited to pronominal subjects or can extend as well to nominal subjects. |
| **F[11-12]-W[74-75]** | English expresses both situational and epistemic possibility using the verbs may, must, and can. **Situational Possibility** refers to the distinctions between “You *may leave* now,” “You *must leave* the hospital after a week”, and “One *can get to* Staten Island using the ferry.” **Epistemic possibility** refers to the distinction between “John may have arrived” and “John must have arrived.” Of the languages that mark epistemic possibility using verbal markings, almost all (98.3%) also mark situational possibility using verbal markings. However, of the languages that mark epistemic possibility using affixes, only some (55.9%) also mark situational possibility using affixes. |
| **F13-W76** | In many languages **epistemic and situational possibility have overlapping markings** (whether shared affixes or shared verbal constructions). This is most common for Indo-European languages. For example, English uses “may” and “must” for both types of possibility, compare “You *may* go home” versus “You *must* go home” (situational) and “Bob *may* be mistaken” versus “Terry *must* be from Northumberland” (epistemic). |
| **F14-W77** | **Evidentials** express the nature of the evidence for a given statement and contrast, e.g., whether the speaker is quoting someone or learned the information second-hand. The evidentiality analysis contrasts languages with no inflectional coding of evidentials with languages that express indirect and/or direct evidentials. Indirect evidentials primarily comprise quotatives (e.g., a morphological encoding for “They say that…”). Direct evidentials contrast different types of direct experience such as knowing by hearing versus knowing by smelling. |
| **F16-W34** | Marking of **plurality on nouns** may either be obligatory or optional, or entirely unavailable. We further break down the optional markings by whether the marking is lexical or inflectional. |
| **F17-W36** | An example of a language with an **associative plural** is Japanese: “Tanaka-tachi” – ‘Tanaka and his associates’. The English “y’all” sometimes functions as an associative plural. |
| **F18-W92** | **Polar questions** are those that elicit a “yes” or “no” response. Many languages have dedicated markers to signal that the sentence is a polar question. WALS codes the position in the sentence of the question particle (which may in some cases be an affix or clitic) as well as its absence, but for the present analysis we collapse across position. An example of a language with a question particle is French: “*Est-ce-que* le président vient?” (Is the president coming?). |
| **F21-W65** | The **perfective** aspect denotes an event conceived as a unit and corresponds to the English past tense. The **imperfective** represents an event in the process of unfolding. Russian: “Ya vypil chai” (I drank the tea until it was gone”) versus the imperfective: “Ya pil chai” ( “I was drinking/drank the tea”). The English use of the progressive to mark the imperfective aspect is a common strategy, but because it does not comprise a dedicated imperfective aspect marker it is not coded by WALS as such. |
| **F22-W70** | Many languages have dedicated morphemes for encoding **imperatives**. Such encoding can differentiate between singular and plural, e.g., Sleep! (singular) versus Sleep! (all of you), or be used only for singular subjects, or have a marker that does not differentiate between singular and plural subjects. There are 2 languages that have a dedicated marker for plural, but not singular subjects (Latvian and Apuriña)—we excluded this category due to the extremely small sample. |
| **F23-W57** | **Possessives** affixes are defined by WALS as those that refer to person, number, and/or gender of a possessor and appear on a noun denoting the entity which is possessed. The term as used in WALS denotes a range of meanings that is broader than that associated with the word possession in English, including not only a relationship of ownership or possession in the everyday sense, but also kinship relations (“my father”), part-whole relations (“my hand”) and other relations as in “its population”, “its foreign minister”, “his first novel”, and “his favourite movie”. A language with a posessive suffix would express the clause “my hand” as hand+[1^st^ person posessive]. |
| **F24-W59** | Many languages morphologically distinguish between various forms of posession, the most common being an alienable/inalienable distinction. For example, it is not uncommon for languages to distinguish between alienable and inalienable possession, e.g., Navajo: bi-be' (her [breast] milk) versus. be-'a-be' (her [store-bought] milk) |
| **F25-W73** | The **optative** refers to an inflected verb form dedicated to the expression of a wish of the speaker. One way to express the optative in English is through modal verbs. The paradigmatic English example is the construction “May God help you.” |
| **F27-W41** | **Distance distinctions** are a spatial analogue of remoteness distinctions. Just as some languages encode different levels of temporal remoteness, so some language differentiate between levels of spatial distance in its demonstratives. English has two levels of contrast, differentiating between “here” and “there.” The term “yonder” to mean “way over there” is now rarely used. Languages with more contrasts frequently differentiate between distance to the speaker versus the hearer. Using Hausa as an example: *nân* (near speaker); *nan* (hear hearer); *cân* (away from speaker and hearer); *can* (far away from speaker and hearer). |
