# Supplementary material for: Language Structure Is Partly Determined by Social Structure
Source: PLoS One. 2010 Jan 20;5(1):e8559. doi: 10.1371/journal.pone.0008559 (PMC2798932; doi:10.1371/journal.pone.0008559)
Supplement: Text S7 — A note regarding multilingualism. (0.02 MB DOC) [file pone.0008559.s011.doc]

**Text S7**

We are aware that multilingualism—often required by exogamy—is the norm in many communities (46). Our argument does not assume that the esoteric linguistic niche consists primarily of monolingual individuals. However, such multilingual environments commonly result from children, rather than adults, learning multiple languages (e.g., a child learning both the mother’s and father’s native languages) (46, 47).
