# Supplementary material for: Language Structure Is Partly Determined by Social Structure
Source: PLoS One. 2010 Jan 20;5(1):e8559. doi: 10.1371/journal.pone.0008559 (PMC2798932; doi:10.1371/journal.pone.0008559)
Supplement: Text S8 — A note regarding generational transmission of a nonnative language. (0.03 MB DOC) [file pone.0008559.s012.doc]

**Text S8**

It has been argued that there is no automatic transmission of the “mother tongue” from parents to offspring (47). For example, in a survey of 188 individuals in Senegal who listed Bambara as their native language, Bambara was the father’s native language in 16%, the mother’s in 19%, the native language of both parents in 26%, and the native language of neither parent in 39% (47). Although children are learning these languages from a young age and are, in theory, fully capable of learning whatever inflectional system the language possesses, in such multilingual environments much of children’s input may come from nonnative speakers. Thus, whatever aspects of Bambara were difficult for the parents to learn are more likely to be passed on to the offspring in a revised form. Although infants clearly generalize beyond their input, if a particular morphological distinction is simply absent from the parents’ input (making their language less specified, but not necessarily ungrammatical), it is less likely to be passed on to the next generation.
