# Supplementary material for: Language Structure Is Partly Determined by Social Structure
Source: PLoS One. 2010 Jan 20;5(1):e8559. doi: 10.1371/journal.pone.0008559 (PMC2798932; doi:10.1371/journal.pone.0008559)
Supplement: Text S9 — A clarification of the term “redundancy.” (0.02 MB DOC) [file pone.0008559.s013.doc]

**Text S9**

“Redundancy” means many different things. As used in the main text, redundancy refers to the degree that grammatically encoded information specifies something that can be readily extracted from context or pragmatics. For example, Russian requires verbs in the present tense to be marked for the gender of the subject (even in first-person discourse). This information is redundant because it is easily obtainable from context/pragmatics. This notion of redundancy was famously satirized by Mark Twain in his essay “The Awful German Language”:

A German speaks of an Englishman as the Engländer; to change the sex, he adds inn, and that stands for Englishwoman -- Engländerinn. That seems descriptive enough, but still it is not exact enough for a German; so he precedes the word with that article which indicates that the creature to follow is feminine, and writes it down thus: "die Engländerinn," -- which means "the she-Englishwoman." I consider that that person is over-described [1].

1. Twain M (2008) The Awful German Language in A Tramp Abroad. Quill Pen Classics.
