# Supplementary material for: Language Structure Is Partly Determined by Social Structure
Source: PLoS One. 2010 Jan 20;5(1):e8559. doi: 10.1371/journal.pone.0008559 (PMC2798932; doi:10.1371/journal.pone.0008559)
Supplement: Text S10 — Modeling language fitness as a function of age of acquisition. (0.56 MB DOC) [file pone.0008559.s014.doc]

### Text S9: Modeling Language Fitness as a Function of Age of Acquisition

Using a simple formal model, Nowak, Komarova, and Niyogi [2] represent a language as a matrix of form-meaning mappings. Meanings are represented as rows of this matrix, and forms/morphemes as columns (Fig. A). Nowak et al. have used such formalisms to explore diverse aspects of language. Poverty (*P_L_*) can be defined as the percentage of grammatical distinctions that are not differentiable from the linguistic signal (Figure A) (e.g., the English pronoun “they” does not encode gender; the difference between they_FEM_ and they_MASC_ generally needs to be deduced from context).

As *P_L_* increases, meanings become less specified by the linguistic signal resulting in simpler form-to-meaning mappings, but creating more ambiguity , requiring a hearer to rely more on extralinguistic context and pragmatic cues. Conversely, as *P_L_* decreases, meanings become more specified by the linguistic signal creating less ambiguity, but at the cost of a more complex form-to-meaning mapping. In terms of Minimum Description Length frameworks [3,4], greater ambiguity corresponds to a greater reconstruction error. We define a new term, redundancy (*R_L_*) which for simplicity varies inversely with *P_L_*.

In this analysis we derive a fitness landscape that is a function of *P_L_* and *R_L_* for languages that vary in their makeup of native (L1) versus non-native (L2) adult learners. This analysis is based on two assumptions. First, we assume that adult L2 learners tolerate a higher reconstruction error than infants due to their more sophisticated ability to rely on extralinguistics/pragmatic cues to meaning. Second, we assume that adult L2 learnres are less capable of learning highly overspecified (low *P_L_*) languages (which in practice, translate to languages with rich morphological systems).


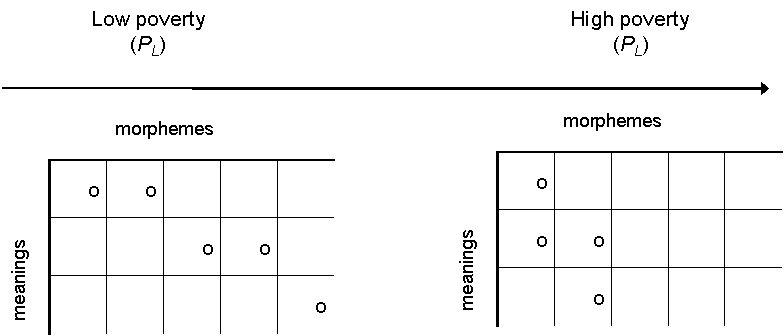


Figure A. Meaning-morphemes mappings with examples of overspecificity and poverty.

We define the learnability of a language *l_L_* given some function of a learner’s age (assuming an errorless process). By assuming two “modes” of learning, L1 and L2, we begin with:


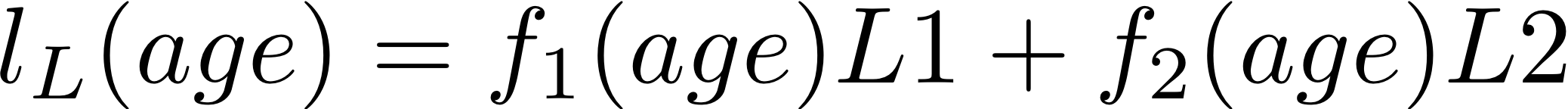


If redundancy (multiple cues to meaning; *R_L_*) helps early acquisition (L1), and poverty, (simpler form-to-meaning mappings; *P_L_*) facilitates adult acquisition (L2) then *l_L_*(*age*), which ranges from 0 to 1, can be represented as a combination of linguistic properties that affect the learnability of a language by speakers of different ages (L1 versus L2 learners). For formal simplification, we represent age as ranging from 0 to 1. In the above equation, *l_L_*(*age*) is defined as the linear combination of two sources of constraint. On the one hand, there is a contribution of the first-language learner’s abilities (L1). When age is small, *f_1_*(*age*) should be near 1 (invoking the L1 constraint) and *f_2_*(*age*) should be near 0. On the other hand, the reverse is true when age is greater: *f_1_*(*age*) should be near 0 (nullifying L1 learning constraints), and *f_2_*(*age*) should be near 1. With this basic intuition, we represent that in the equation below where *M* is a constant to scale the learnability function such that d*beta’*(*M*)/dx = 0:


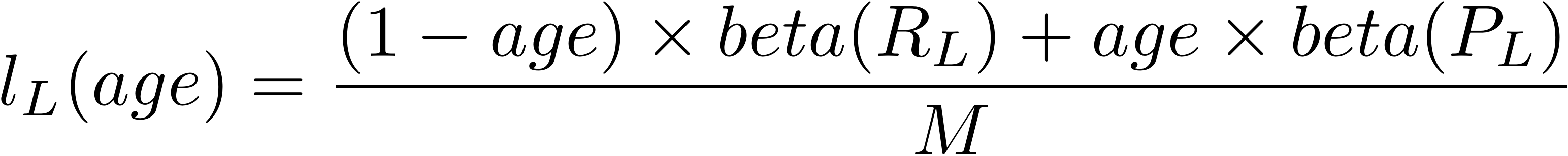


This equation satisfies the general form expected from the combination of L1/L2 constraints just described. Because age is represented in the range of 0 to 1, we can weight the application of poverty and redundancy by *age* and 1 *– age* where appropriate. In the latter case, when age is small, 1 *– age* is greater, thus invoking the *beta*(*R_L_*) function. Here, *beta* is the beta distribution,


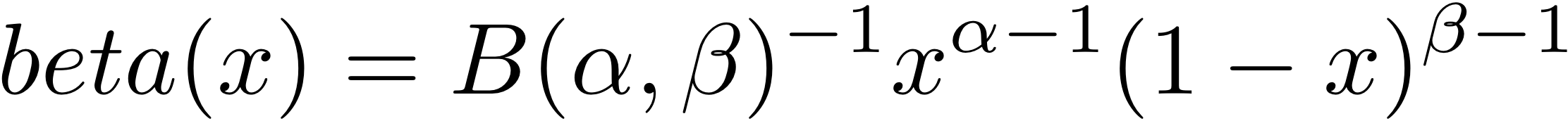


This beta function is visualized in Fig. B. We chose *α* = 4, and *β* = 2, to capture the fact that 100% *O_L_* and *P_L_* would diminish learnability, and there is an ideal intermediate value for each property (e.g., absolute poverty would be absent of any encoded meanings).

Figure B: The contribution of overspecificity and poverty to the learnability function. This nonlinear function is scaled by age to determine how redundancy, *R_L_*, and poverty, *P_L_*, separately specify learnability for a language comprised of infant (L1) versus adult (L2) learners.

These beta functions are scaled by age to formalize the assumption that adults, due to their greater ability to reconstruct meanings from partial/underspecified messages, require less specificity to recover a meaning from a message.

As in [2], we define the overall fitness of a language *F_L_* as the expected learnability across a population. In practical terms, this would represent a sample *S* of learners from a population, determining their age of acquisition, and summing the individual *l*_L_’s to compute the overall learnability of a given language by its population:


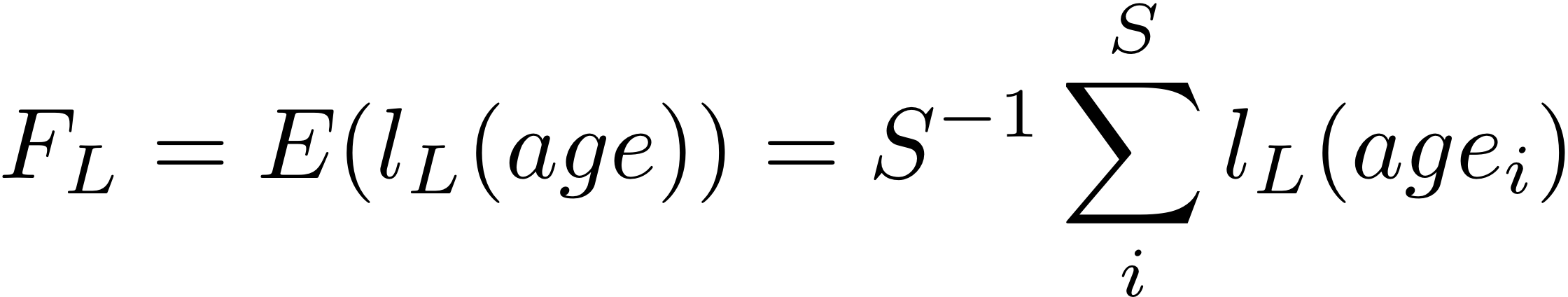


Here, *age_i_* reflects the age at which acquisition took place. If we assume fixed values for R*_L_* and *P_L_*, the above expression may be expanded through *l_L_*(*age*), and fitness becomes a function of the average age of acquisition for that population:


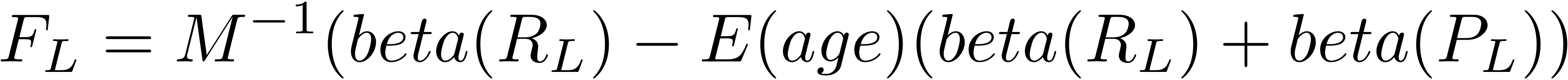


Under the assumption that P_L_ and R_L_ are inversely related, we impose the very simple relationship *R_L_* = 1 *- P_L_*. We now apply the above equation for varying values of *P_L_*  and *R_L_* to languages that are composed of varying proportions of L1 and L2 learners. We generate various proportions by sampling over a population with L1 acquisition age as 0 and L2 acquisition age as a sampling over a random uniform distribution between .5 and 1.

Figure C shows the fitness contours for languages with varying proportions of L1 to L2 learners. For languages with high proportions of L2 to L1 learners, maximum fitness is obtained by minimizing grammatical distinctions (high *P_L_*, ), and by extension, less redundancy (*R_L_*). For languages with low proportions of L2 to L1 learners, maximum fitness is obtained with more complex form-to-meaning mappings.


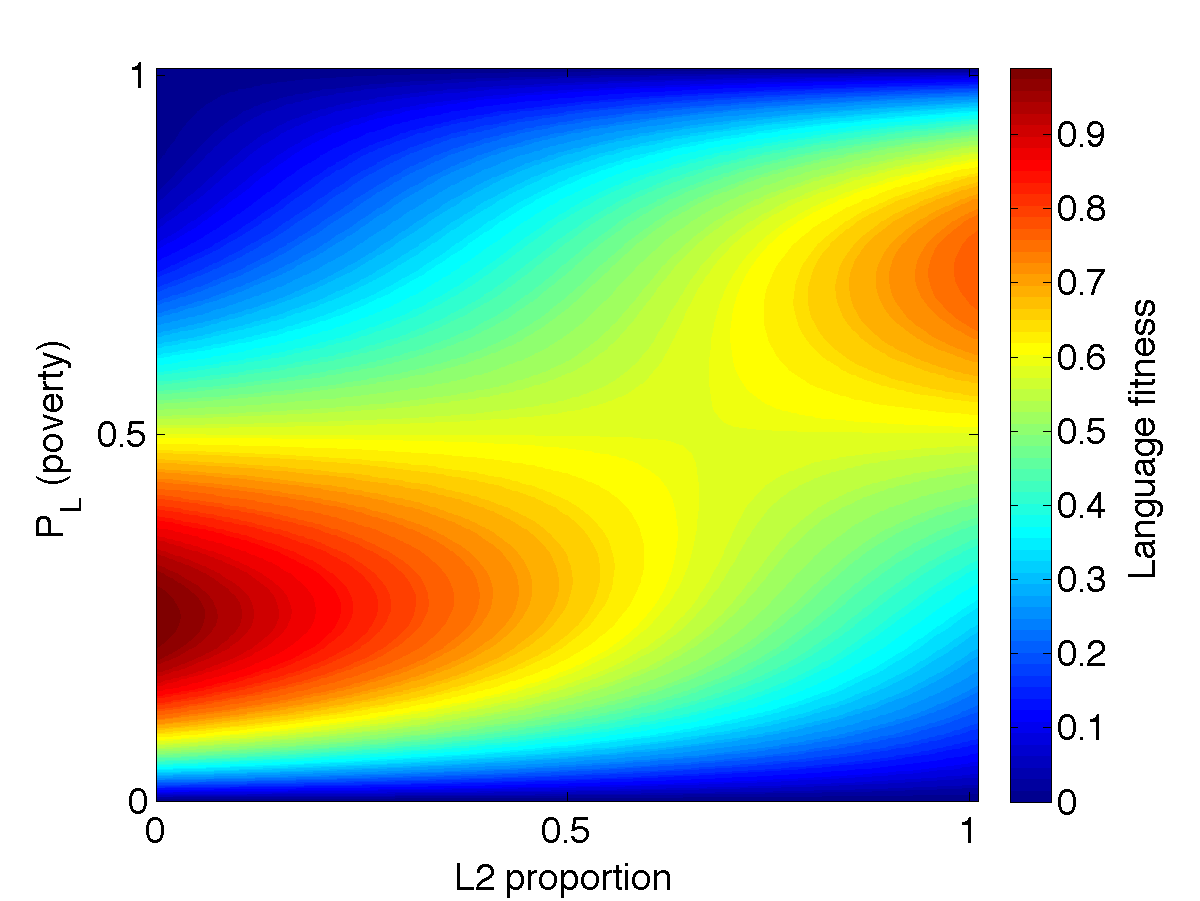


Figure C: The tradeoff between poverty and overspecificity across proportion of L2 speakers. As the proportion of L2 learners increases, greatest language fitness is obtained for languages that minimize grammatical distinctions.

Although L1 learners are unconstrained by the number of grammatical distinctions (*P_L_*), they are constrained by ambiguity, requiring more redundant languages (which have correspondingly low P_L_ values). Conversely, adult L2 learners can learn languages of any ambiguity, but are constrained by the number and complexity of grammatical distinctions (*P_L_*).

It may be argued that although L2 acquisition is constrained by the complexity of form-to-meaning mappings (P_L_), L1 acquisition is robust under any conditions and is not constrained by redundancy (Figure D).


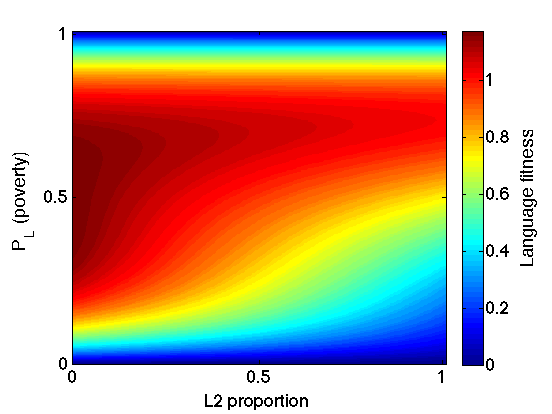


Figure D: A fitness function when language learning is unconstrained by redundancy.

To test whether redundancy in languages varies as a function of the learning population, we conducted an analysis aimed at quantifying compressibility of text written in different languages as a function of the language population (using overall population as a proxy measure for L2 acquisition, as used in the main text).

2. Nowak MA, Komarova NL, Niyogi P (2002) Computational and evolutionary aspects of language. Nature 417: 611-617. doi:10.1038/nature00771

3. Rissanen J (1983) A universal prior for integers and estimation by minimum description length. Annals of Statistics 11: 416-431.

4. Zemel R, Hinton G (1995) Learning Population Codes by Minimizing Description Length. Neural Comput 7: 549-564.
