# Supplementary material for: Language Structure Is Partly Determined by Social Structure
Source: PLoS One. 2010 Jan 20;5(1):e8559. doi: 10.1371/journal.pone.0008559 (PMC2798932; doi:10.1371/journal.pone.0008559)

### Text S10: Text Compressibility as a Measure of Linguistic Redundancy

As a test of the prediction that languages spoken in the esoteric niche (i.e., those with few L2 learners) may be more informationally redundant than languages spoken in the exoteric niche, we obtained written translations of the same text (Universal Declaration of Human Rights [5]) for languages that use the Roman Alphabet (N=103). Redundancy was assessed by compressing the text files corresponding to each language using a standard ZIP algorithm (as implemented by WinRAR: www.rarlab.com). Overall, we expected that greater morphological specification would lead to greater compressibility. For example, the strings “walk” and “walked” can be compressed by storing “walk” and “ed” in a dictionary and referencing “ed” for any regularly inflected verb, producing a storage savings whenever an inflected verb occurs (of course the addition of inflections can increase the overall size of the uncompressed document). In the absence of an inflectional past tense marker, no such savings occurs.

Table S2 shows the obtained correlations between the measure of redundancy (compression ratio) and the demographic variables used in our main analysis. Supporting Figure 1 shows the relationship between population and compressibility of the text. We found that languages spoken by more people and/or over a larger area are less compressible than languages spoken by fewer people (Figure S1). A set of control analyses were run to demonstrate that it is indeed morphological coding that generates this compressability pattern across languages (Table S2).

Because longer documents result in more complete dictionaries (asymptotically reaching the optimal compression ratio for a given language), compression ratios are positively correlated with original file size and negatively correlated with the number of unique words. Additional analyses that partialed out the original file size and the number of unique and total words, did not eliminate the negative relationship between population and compressibility. To ensure that the redundancy differences arose from differences in morphological specification, we replaced each unique word with a unique number, e.g., “walk” and “walked” might be consistently replaced throughout the document by the numbers “12938” and “59843.” [6]. This substitution retains the relationship between words (each repeated occurrence of “12938” can still be replaced by a reference to the dictionary entry), but not between morphemes. Following the substitution, there were no significant relationships between demographics and compressibility suggesting that the original relationship was driven by morphology.

The present finding that compressibility (redundancy) varies as a function of language population is compatible with a language fitness function in Figure C and provides evidence against the function depicted in Figure D in which language-learning is unconstrained by redundancy.

5. Universal Declaration of Human Rights (n.d.). Available: http://www.un.org/.

6. Juola P (1998) Measuring Linguistic Complexity: The Morphological Tier. Journal of Quantitative Linguistics 5: 206-213.

### Table: Pearson correlations (and p-values) for the compression (ZIP) analysis of text translations of the Universal Declaration of Human Rights into 103 languages.

|  | | Population  (Log Speakers) | Area  (Log km^2^) | Ling Contact  (Log ling. neighbors) |
| --- | --- | --- | --- | --- |
| **Total Words** | | -.01 | -.17  (.10) | -.11 |
| **Unique Words** | | .15  (.14) | .17  (.10) | .04 |
| **Size in bytes** | | **-.23**  (.02) | -.19  (.06) | -.12 |
| **Compression Ratio (CR)** | | **-.56**  (<.0005) | **-.32**  (<.0005) | -.17  (.09) |
| **CR with size partialed out** | | **-.43**  (<.0005) | **-.22**  (.03) | -.10 |
| **CR with total and unique words partialed out** | | **-.53**  (<.0005) | **-.25**  (.01) | -.13 |
| **Control**  **Condition** | | | | |
| ***CR With Number Substitution*** | -.01 | | -.12 | -.03 |

Figure. Text translated into languages spoken by fewer people is more compressible (i.e., more redundant), compared to text translated to languages spoken by many people.


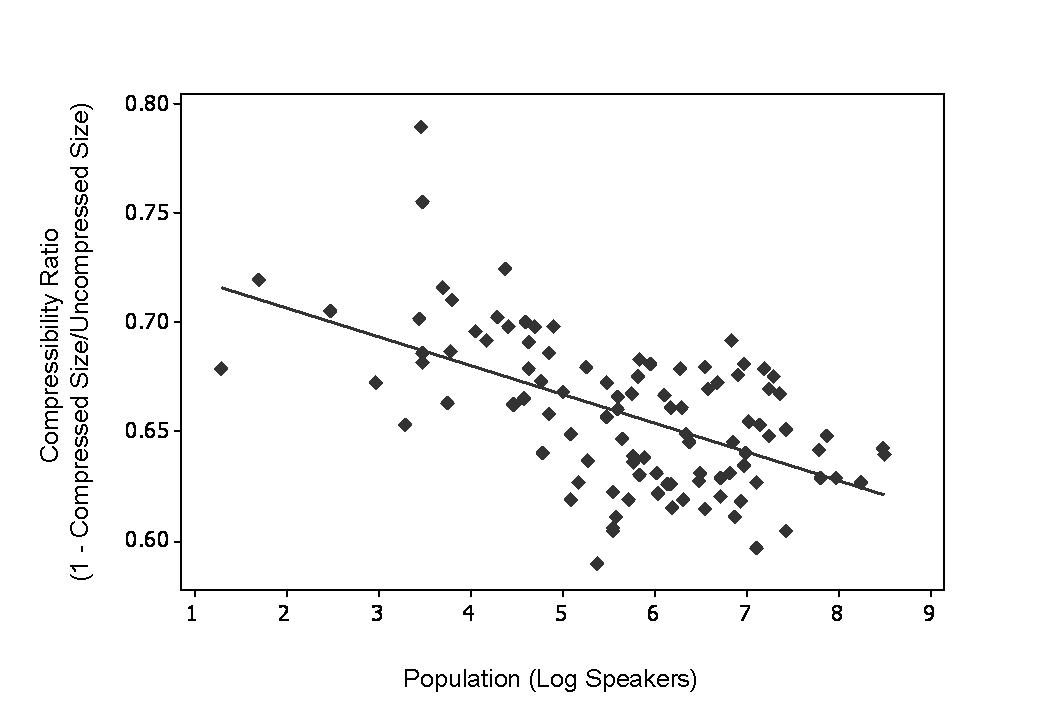

Supplement: Text S11 — Text compressibility as a measure of linguistic redundancy. (0.10 MB DOC) [file pone.0008559.s015.doc]
