# Supplementary material for: Language Structure Is Partly Determined by Social Structure
Source: PLoS One. 2010 Jan 20;5(1):e8559. doi: 10.1371/journal.pone.0008559 (PMC2798932; doi:10.1371/journal.pone.0008559)
Supplement: Text S12 — Supporting analyses of constituent order and application to adult versus child language acquisition. (0.04 MB DOC) [file pone.0008559.s016.doc]

### Text S11: Supporting Analyses of Constituent Order and Application to Adult versus Child Language Acquisition.

Our analyses in the main text have shown that languages that are spoken in the exoteric niche have simpler inflectional morphology, and more broadly, are less grammatically specified than languages spoken in the esoteric niche. In this Supporting analysis we show that languages spoken in the exoteric niche often exhibit constituent ordering that deviates from the dominant patterns observed among the world’s languages. For example WALS [7] feature 26 (http://wals.info/feature/26) classifies languages into those that use prefixing versus suffixing in inflectional morphology. Of 772 languages in the databases that have affixes, 382 are classified as being “strongly suffixing” (representing 66 language families). In contrast, only 54 languages (14 families) are classified as being strongly prefixing. The latter are absent from Eurasia, but are not confined to any one part of the world, occurring in Africa, The Pacific, and the Americas. The frequency of occurrence of a linguistic feature or type such as suffixing is often taken as an indication of its greater naturalness or learnability [8]. On this view, languages adapt to the learning biases of the human brain and typological asymmetries occur because some linguistic devices are easier to acquire or provide a communicative advantage. The present formulation of the linguistic niche hypothesis *requires one to specify whom the greater learnability is benefiting*. For languages learned exclusively by infants, greater typological frequency may indeed correspond to e.g., ease of first language acquisition. However, insofar as children and adults may have different learning constraints and biases, languages adapted to the exoteric niche (as indicated by larger populations, and greater spread and linguistic contact), may diverge from the allegedly most learnable patterns exemplified by the most frequent typologies. Indeed, even though inflectional suffixing is far more common than inflectional prefixing, the speaker population of languages that use inflectional prefixes exceed those that use inflectional suffixes by a factor of 10, *F*(1, 413)=23.19, *p*<.0005—a pattern mirrored by a difference in the degree of linguistic contact, with prefixing language having more neighbors than suffixing languages, *F*(1, 390)=12.57, *p*<.0005 (GLMs controlling for geographic location) (Figure S2 a-b). This suggests that whereas inflectional suffixes may be most natural for infant learners, prefixes may be more natural for adult learners. There is strong evidence in the child language acquisition literature for earlier acquisition of suffixes than prefixes [9-11] and some evidence that adults learn prefixes more easily than suffixes in artificial language-learning tasks [12,13]. It may be of interest that the one language in South America to use inflectional prefixes is Guaraní—a language of Paraguay—which has served as a lingua franca over large parts of Brazil and is perhaps the only South American indigenous language to have mostly non-indigenous speakers.

Table S1 contrasts patterns related to constituent order that are exemplified by languages occupying the exoteric niche with the most frequently observed patterns. Unlike our primary analyses which focused on morphological complexity, this Supporting analysis does not lend itself to *a priori* predictions regarding relative complexity of the listed variants, e.g., it is not clear whether suffixes or prefixes are more complex. Hence, we did not make predictions of which constituent order would be associated with languages adapted to the exoteric niche. However, comparing the pattern common to exoteric-niche languages with the most widespread patterns, allows us to make predictions regarding the relative naturalness of constituent order for child learners (who constrain all languages) and adult learners (who constrain languages spoken in the exoteric, but not esoteric niche). With one exception, the features listed in Table S1 make different predictions for the relative naturalness of constituent orders (the one exception is the ordering of nouns relative to degree modifiers such as “very”). For example, subject-object-verb (SOV) is the most widespread basic word order and would thus be predicted to be the most natural. Yet, SVO languages have significantly more speakers, suggesting that as linguistic structure becomes constrained by adult acquisition, a language is more likely to exhibit SVO ordering (Figure S2 c-d). The predictions can be tested using artificial language tasks as well as in natural language learning studies that contrast child and adult learners. It is noteworthy that Pidgin and Creole languages—those that have been most shaped by adult acquisition—not only lack complex morphology and case markings, but with few exceptions, have an SVO order [14].

It is perhaps noteworthy that morphologically complex languages often tend to have simpler syntax, particularly with respect to embedding [for review, see 15]. If there is indeed such a tradeoff—complex morphology perhaps allowing for simpler embedding—one can ask whether languages unconstrained by adult learning biases are more likely to have simpler embedding in part due to children’s relative difficulty with syntactic emedding compared to acquiring morphology for expressing the same information. This is an area awaiting empirical study.

7. Haspelmath M, Dryer M, Gil D, Comrie B (n.d.) The world atlas of language structures online. Munich: Max Planck Digital Library.

8. Christiansen M, Chater N (2008) Language as Shaped by the Brain. Behavioral and Brain Sciences 31: 489-509.

9. Slobin D (1979) Psycholinguistics. 2nd ed. Scott Foresman & Co.

10. Slobin D (1985) Crosslinguistic evidence for the language
making capacity. In: Slobin D, editor. The crosslinguistic study of language acquisition. Vol 2: Theoretical Issues. Hillsdale, NJ: Erlbaum. pp. 1157-1256.

11. Kuczaj S (1979) Evidence for a Language Learning Strategy: On the Relative Ease of Acquisition of Prefixes and Suffixes. Child Development 50: 1-13.

12. Frigo L, McDonald JL (1998) Properties of Phonological Markers That Affect the Acquisition of Gender-Like Subclasses, , Journal of Memory and Language 39: 218-245. doi:10.1006/jmla.1998.2569

13. MacWhinney B (1983) Miniature linguistic systems as tests of the use of universal operating principles in second-language learning by children and adults. Journal of Psycholinguistic Research 12: 467-478. doi:10.1007/BF01068027

14. McWhorter J (2001) The world’s simplest grammars are creole grammars. Linguistic Typology 5: 125-166.

15. Evans N, Levinson S The Myth of Language Universals: Language diversity and its importance for cognitive science. Behavioral and Brain Sciences. in press.

16. Gordon RG (2005) Ethnologue: Languages of the World, 15th Edition. Fifteenth. SIL International.
